# Promiscuous class II-binding SARS-CoV-2-nuc derived vaccine-peptide induced extensive conventional, innate and unconventional T cell responses

**DOI:** 10.3389/fimmu.2025.1676455

**Published:** 2025-11-11

**Authors:** Naomi Krickeberg, Hans-Georg Rammensee, Karin Schilbach

**Affiliations:** 1 Department of Pediatric Hematology and Oncology, University Children’s Hospital Tübingen, Tübingen, Germany; 2 Institute of Immunology, University of Tübingen, Tübingen, Germany; 3 German Cancer Consortium (DKTK), partner site Tübingen, a partnership between DKFZ and University Hospital Tübingen, Germany, Cluster of Excellence iFIT (EXC2180) “Image-Guided and Functionally Instructed Tumor Therapies”, University of Tübingen, Tübingen, Germany

**Keywords:** SARS-CoV-2, peptide vaccine, SARS-CoV-2 nucleoprotein peptide, T cell response, unconventional T cells, adaptive Vδ2γ9^negative^ γδ T-cells, CD1 restricted, vaccine peptide reactive Vδ1 γδ T-cells

## Abstract

We describe the T-cell response of two healthy SARS-CoV-2-unexposed volunteers to a SARS-CoV-2 nucleoprotein-derived vaccine peptide predicted to promiscuously bind multiple HLA-DR allotypes. NGS-based bulk TCR-repertoire analysis of peptide-specific T-cell responses 4 (D2) and 27 (D1) weeks after vaccination identified CDR3 regions of TCRα, -β, -γ and -δ chains in T cells responding *ex-vivo* to the vaccine peptide LLLLDRLNQLESKMS with IFNγ^+^-secretion. Adaptive repertoires were unique. Donors shared 15 TCRα and 9 TCRβ clonotypes, all public, showing no conserved motifs but TdT-independent “neonatal” CDR3 regions close to the germline. Half the wtSARS-CoV-2 nucleocapsid-reactive adaptive clonotypes show preferential V-segment usage (6/64 Vα and 4-8/45 Vβ chains), and all share/show a N-nucleotide-encoded hydrophobicity in their CDR3 region. VδCα rearrangements (20.4% and 15.3% of the TCRα-repertoires, respectively), Vδ1Cδ γδ-clonotypes homologous to public CD1-restricted Vδ1^+^ γδTCRs, and the induction of “adaptive” Vδ2Vγ9^negative^ T cells support the role of innate T cells in the immune response.

## Introduction

SARS-CoV-2, the causative agent of coronavirus disease 19 (COVID-19), has caused devastating global morbidity and mortality (1) particularly in the elderly (2), people with chronic diseases (3), and co-morbidities such as cancer (4).

### Importance of T cell immunity

Globally licensed COVID-19 vaccines focus on humoral immune responses as protective immune mediators (5). However, short duration and limited neutralizing effect against certain virus variants provide only temporary protection and require repeated vaccine boosters. T-cell induction is essential for survival, but specific information on the role and potency of T-cell responses in Covid-19 exposed individuals is still limited. The paramount importance of T cells is demonstrated by the fact that in primary infection B cells rely on a robust response from follicular CD4^+^ T helper cells to develop neutralizing antibodies (6). In contrast, T cells can efficiently control SARS-CoV-2 infection even in the absence of a humoral response (7–13).

### Role of memory CD4^+^ T cells

CD4^+^ T cells orchestrate the antiviral adaptive immune response by enhancing CD8^+^ T cell and B cell responses. Memory T cells are the first line of defense against reinfection and generate an immunological memory that can provide lifelong protection against pathogens. Recent studies have shown that SARS-CoV-1 T-cell immunity persists for decades (14) and that virus-specific memory T cells can persist for up to 75 years in humans (15). In addition, it is increasingly recognized that CD4^+^ T cells play an essential role in the control of chronic viral infections (16–18). The development of vaccines that induce effective, and in particular CD4^+^ T-cell responses, is therefore highly attractive.

### Objective of the present study

Rammensee et al. designed SARS-CoV-2 HLA-DR T-cell epitopes specifically for their promiscuous binding to multiple HLA-DR alleles covering 97% of the Western population (19). That these peptides were derived from different viral proteins is particularly important because unbiased screens show that T cells from COVID-19 patients recognize SARS-CoV-2 epitopes largely outside the spike protein (20, 21).

Here, we evaluate SARSCoV-2-nuc derived LLLLDRLNQLESKMS peptide – designed for promiscuous binding of HLA class II - for its potential to induce a (CD4^+^) T-cell response (19). LLLLDRLNQLESKMS was applied - emulsified in Montanide – with Toll-like receptor (TLR) 1/2 agonist XS15 to provide potent activation and maturation of antigen-presenting cells and to prevent vaccine peptides from immediate degradation, thereby supporting the induction of a potent T-cell response (16). Immune responses were assessed in 2 elderly healthy SARS-CoV-2-unexposed (non-infected, non-recovered) volunteers, both vaccinated in the course of a self-experiment. LLLLDRLNQLESKMS-specific T-cell clones were identified *ex vivo* by peptide-induced cytokine secretion and subjected to TCR-bulk sequencing.

In addition to conventional αβ-T cells, CD1-reactive Vδ1^+^ and adaptive Vδ2^+^ γδ-T cells, as well as Vα/Cδ- and Vδ/Cα clonotypes, were induced. Characteristic features of the immune cell phenotypes responding to the vaccine-peptide, specifically designed for promiscuous binding to HLA class II are shown and discussed.

## Materials and methods

### Vaccinated individuals

Vaccinated individuals were two healthy, noninfected, non-convalescent volunteers age ≥65. Their HLA class II haplotypes were HLA-DRB1*11:01 (Donor1), and HLA-DRB1*01:01 and HLA-DRB1*11:0401 (Donor2) respectively. For T cell responses Donor 1 was analyzed 4 weeks and Donor 2 five months post inoculation with SARS-CoV-2 nuc Peptide. Vaccination was performed with the synthetic peptide solubilized in water and 20% DMSO including 50 μg XS15 as an adjuvant, emulsified in Montanide ISA51 VG in a total volume of 0.5 mL. The vaccine was administered as a single subcutaneous injection in the left side of the abdomen.

*Why targeting the nucleoprotein:* The SARS-CoV-2 spike protein exhibits a markedly higher mutation rate than other viral proteins, estimated at approximately 8 × 10^−4^ substitutions per site per year, which is around 3–5 times higher than the overall genome average (22, 23). This accelerated evolution drives the emergence of variants with over 30 significant mutations in spike alone, contributing to immune evasion and challenges to vaccine effectiveness (24). In contrast, the nucleoprotein (N) of SARS-CoV-2 is highly conserved with around 90% amino acid sequence identity to SARS-CoV-1 (25) compared to approximately 76% for spike (26), making N a stable target for T cell immunity. Moreover: N is produced at high levels in infected cells, making it an important marker for infection (27). Together, these data justify the choice of nucleoprotein-derived peptides in vaccine design to complement spike-targeted approaches by providing broader and more durable T cell immunity against SARS-CoV-2.

*As for the adjuvant XS15*: a synthetic, water-soluble derivative of the TLR1/2 ligand Pam3Cys, developed as a peptide vaccine adjuvant by Rammensee et al. (28) activates TLR1/2 heterodimers, stimulating dendritic cells to produce proinflammatory cytokines (IL-8, MCP-1, MIP-1β) and upregulate activation markers (HLA-DR, CD83, CD86). When admixed with peptides (without covalent coupling), XS15 induces strong CD4^+^ Th1 polarization and CD8^+^ T-cell responses. Its solubility and GMP suitability render it ideal for personalized peptide vaccines. Clinical trials in AML (29), CLL (30), and COVID-19 in B cell deficient patients (31) demonstrated safety and durable immunogenicity, making XS15 a promising adjuvant for personalized tumor and infectious disease vaccines.

### Vaccine-peptide LLLLDRLNQLESKMS

#### Reasoning for selecting presentation by MHC class II

Promiscuous MHC class II peptides induce broad CD4^+^ T cell responses by binding multiple HLA-DR, -DP, and -DQ alleles, greatly increase the likelihood of eliciting helper T cell responses in most individuals, regardless of their HLA genotype (32). CD4^+^ T cells support CD8^+^ cytotoxic expansion, license APCs, differentiate into T follicular helper cells for humoral immunity, and exhibit potent recall responses coordinating cellular and humoral arms (33). Moreover, helper epitopes are essential for neoantigens to elicit effective CD8^+^ responses and reveal antitumor immunity against subdominant MHC I–restricted neoepitopes (34). Moreover distinct from CD8^+^ T cells, CD4^+^ T cells display potent recall responses and provide ongoing immunologic surveillance—often being more robust and durable than CD8^+^ responses in both natural infection and vaccination due to their ability to respond to antigen re-encounter and coordinate both cellular and humoral arms of immunity (35).

SARS-CoV-2 nuc-derived peptide LLLLDRLNQLESKMS predicted to bind to multiple HLA-DR molecules (HLA-DRB1*01: 01, HLA-DRB1*03:01, HLA-DRB1*04:01, HLA-DRB1*07:01, HLA-DRB1*11:01, HLA-DRB1*15:01) (19) was kindly provided by H.-G. Rammensee. A pipeline for the prediction for promiscuous binding to HLA-DR was established and described by Rammensee et al. (36, 37).

### Likelihood of presentation by MHC class I

We also investigated whether shorter variants of this specific peptide are able to bind to a particular HLA class I molecule. For determining HLA restriction computational prediction tools such as NetMHCpan-4.0 were employed (38). The embedded peptide LLLDRLLNQL is predicted to be restricted to HLA-A*02 as a weak binder (0.6% rank) (37), with both donors being negative to this allele.

LLLLDRLNQLESKMS was part of the multi-peptide vaccine CoVac-1, designed to induce broad T cell immunity against SARS-CoV-2 by including promiscuous HLA-DR-restricted peptides derived from multiple viral proteins, described in the recent study by Rammensee et al., published in Nature (39).

As for the likelihood of SARS-CoV-2 nuc descending peptide LLLLDRLNQLESKMS for CD1 presentation we used the Castano CD1d binding algorithm (1-4–7 rule) (**Table 1**), which shows that hydrophobic peptides can be presented by CD1 when hydrophobic aa are present at positions 1-4-7 (40). Mechanistically, amphipathic or hydrophobic peptides can enter a cell through membrane disruption (41–43) and find their way to lipid droplets (44) where they are subjected to CD1 loading (45).

**Table 1:** Vaccine peptide LLLLDRLNQLESKMS.

Peptide	AA position in SARS-Cov-2 nuc	Peptide length
**L**LL**L**DR**L**NQLESKMS Pept.Pos: **1–4–7**(40)	221–235	15

G P A V I L M Aliphatic amino acids, increasing in hydrophobicity from left to right.

Amino acid positions that allow CD1 presentation of lipophilic peptides according to Castano et al. (40) are marked in bold and red.

### Immune monitoring

#### Enrichment of peptide-specific T-cells

Immune monitoring was performed at one timepoint 4 weeks after vaccine administration in Donor 1 and 5 months after the last vaccine administration in Donor 2. PBMCs were isolated from heparinized blood by Ficoll density gradient centrifugation (Biocoll Separating Solution, Bio & SELL) and plated in RPMI supplemented with 5% autologous serum at a density of 1 x 107 cells/ml in a 24-well plate. A total of 6 x 10^7^ PBMCs were plated for each donor respectively. Cells were stimulated with the peptide (*LLLLDRLNQLESKMS*) that was identical in both patient-individualized vaccines. A peptide stimulus was provided on d1 (5 µg/ml), D8 (2,5 µg/ml) and on D15 (2,5 µg/ml). On day 2 cells were split to a density of 5 x 10^6^ cells/ml to avoid bystander activation of T-cells, cells were split again on day 11 to a density of 2,5 x 10^6^ cells/ml. Fresh medium supplemented with 5% autologous serum was provided twice a week. On day 15, cells that after 5h secreted IFN-γ in response to the peptide stimulus were isolated with the IFN-γ Secretion Assay Cell Enrichment and Detection Kit (Miltenyi Biotec) according to the manufacturer’s instructions.

### Flow cytometry

Cells were stained before and after IFN-γ enrichment using the following antibodies CD14-FITC (M5E2, BD Pharmingen), CD3-PerCP (SK7, BioLegend), CD4-VioBlue (VIT4, Miltenyi Biotec), CD8-APC-H7 (SK1, BD Pharmingen). Dead cells were excluded using Zombie Aqua™ Fixable Viability Kit (BioLegend). All cells were acquired on a flow cytometer (BD FACSCanto II, BD Biosciences) and flow cytometry results were analyzed using FlowJo™ v10.8 Software (BD Life Sciences).

### Molecular methods

#### RNA extraction

RNA from IFN-γ enriched cells was extracted using the Quick-RNA Microprep or Miniprep Kit (ZymoResearch) according to manufacturer’s instructions. Quality and integrity of extracted RNA was evaluated on a Fragment Analyzer System 5300 (Agilent) and concentration of RNA was measured on a Qubit 3 Fluorometer (Thermo Fisher Scientific).

### TCR sequencing

#### Library preparation

α/β T cell receptor libraries were prepared using SMARTer Human TCR a/b Profiling Kit v2 (Takara Bio) according to manufacturer’s instructions with a maximum RNA Input of 273,6 ng for D1 and 151,05 ng for Donor 2. To facilitate sequencing of γ/δ TCRs, γ/δ T cell receptor libraries were prepared with the same kit but with a modified protocol including custom γ/δ primers by the commercial service provider MedGenome Inc. Standardized RNA input for γ/δ TCR libraries was 10 ng for each sample respectively.

### NGS sequencing parameters

Sequencing was performed with a MiSeq system (Illumina) using the MiSeq Reagent Kit v3 600-cycle.

### Sequencing data analysis

#### Primary data analysis

The TCR sequencing data was analyzed using the nf-core/airrflow pipeline version 2.1.0 (46), which is an open-source pipeline written in Nextflow (47) and available at http://github.com/nf-core/airrflow as part of the nf-core project (48). The pipeline employs the Immcantation toolset (49–51) for processing of repertoire sequencing data. The sequencing quality of the Illumina MiSeq high-throughput sequencing reads was evaluated with FastQC (52). The pRESTO toolset (49) was used for processing the sequencing reads. Reads were filtered according to base quality (quality score threshold of 20), the forward and reverse reads were paired and a consensus sequence from reads with the same UMI barcodes was obtained, allowing a maximum mismatch error rate of 0.1 per read group. V(D)J sequences were only considered that had at least 2 representative sequences to build the consensus. Sequence copies were calculated as the number of identical sequences with different UMI barcodes. Variable-diversity-joining [V(D)J] germline segments were assigned by aligning the processed sequences to the IMGT database with Change-O and IgBLAST (50, 53). Functional V(D)J sequences were considered part of the same clonal group when they shared the same V(D)J assignment and an identical CDR3. Repertoire characterization was performed with Alakazam and SHazaM (52).

#### Tools used

Logoplots showing amino acid sequence similarity were generated using Glam2 (54).

Post-processing data analysis of TCRseq data was performed using in-house developed R scripts using R statistical software (v4.1.0) (55) and Rstudio (2023.03.0). R packages used included ggplot2 (v3.3.5) (56), packcircles (v0.3.5) (57) for treeplots and circlize (v0.4.13) (58) for chord diagrams for rearrangements.

Clustering of TRB: Clustering of TRB clonotypes was performed using the GLIPH2 (grouping of lymphocyte interactions with paratope hotspots 2) algorithm (59).

Screening for clonotype publicity: Screening for the public nature of a clonotype was performed using the iReceptor Scientific Gateway of the iReceptor platform (60).

### Data availability

The subjects’ repertoire data are publicly available as part of the AIRR Data Commons on VDJ Server (https://vdj-dev.tacc.utexas.edu/) under the permanent identifier c8aa9206-c53f-408b-a517-a74e131547b6. The raw fastq sequencing files have been deposited in NCBI’s Sequence Read Archive (SRA) and are accessible under the BioProject PRJNA1232000 through the SRA accession numbers SRR32574224 and SRR32574225.

### Study design

**Figure 1:**
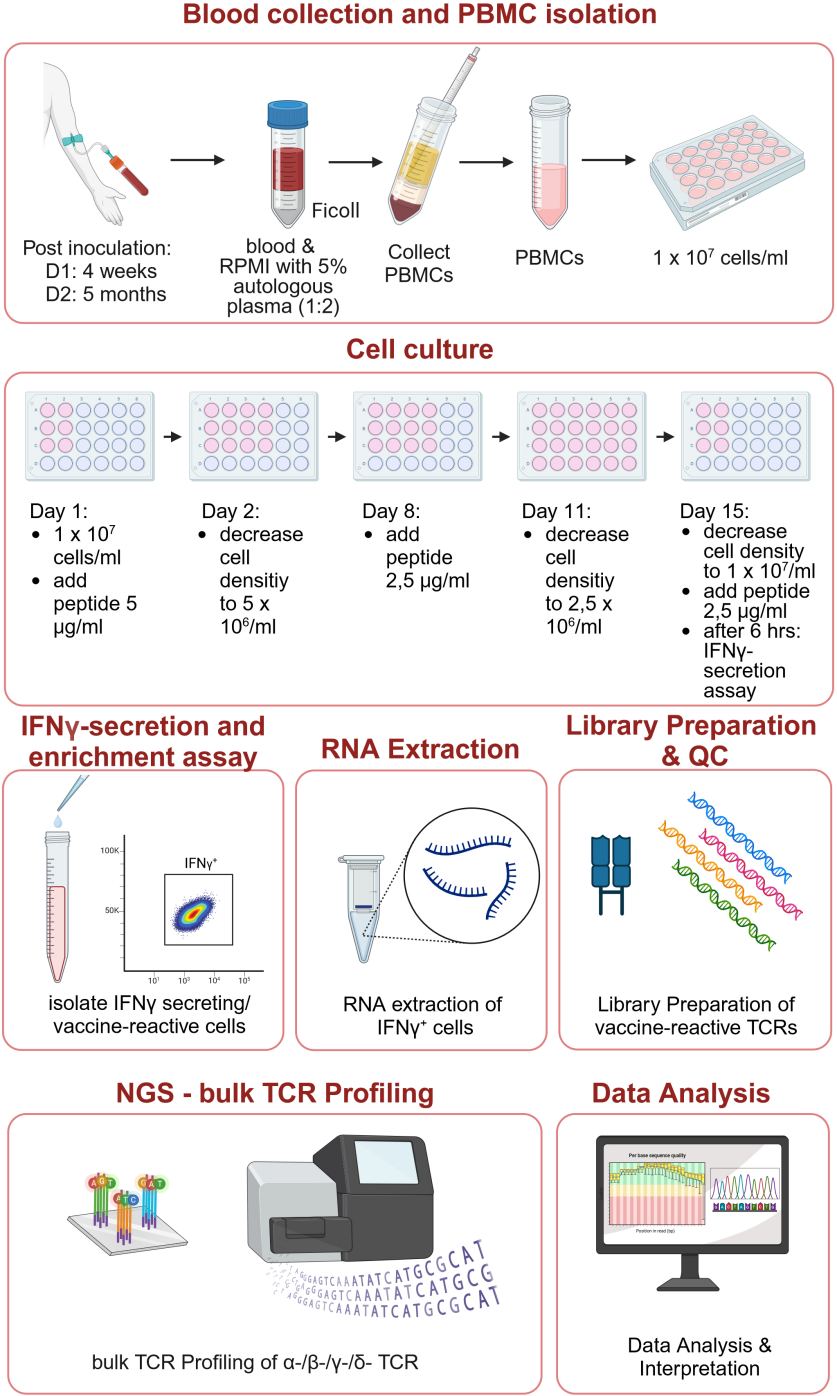
Study design. Created in BioRender. (2024). https://BioRender.com/w63u910.

PBMCs were isolated from freshly drawn heparinized peripheral blood of the two vaccinated individuals. To amplify vaccine-peptide reactive clones, freshly isolated PBMCs were placed in short-term culture ex vivo with selecting conditions so that only peptide specific T cells would survive due to autologous IL-2 production. For culture details see Sonntag et al (61) and MM section below. T cells responding to the vaccine-peptide pulse with IFNγ secretion were harvested on day 15 (IFN-γ Secretion Assay Cell Enrichment and Detection Kit, Miltenyi Biotec), analyzed by flow cytometry for coreceptor/cytokine expression; vaccine reactive T cell´s RNA was extracted and subjected to NGS bulk T cell receptor sequencing (**Figure 1**).

### CDR3 usage for identification/characterization of the immune response

The CDR3-IMGT is delimited in 5′ by the V-REGION 2nd-CYS 104 and in 3′ by the J-REGION position 117. IMGT/Junction Analysis explores the JUNCTION that is delimited in 5′ by the V-REGION 2nd-CYS 104 and in 3′ by the J-REGION J-TRP 118 (for the IGHJ).

### Ethic vote

This study was in accordance with the ethical standards of the institutional ethic committee of the Medical Faculty of the Eberhard-Karl-University Tübingen; approved reference: 757/2021B01.

## Results

### Promiscuously MHC class II-binding SARS-CoV2-vaccine-peptide induced a strong CD4^+^ T-cell response in both recipients

**Figure 2:**
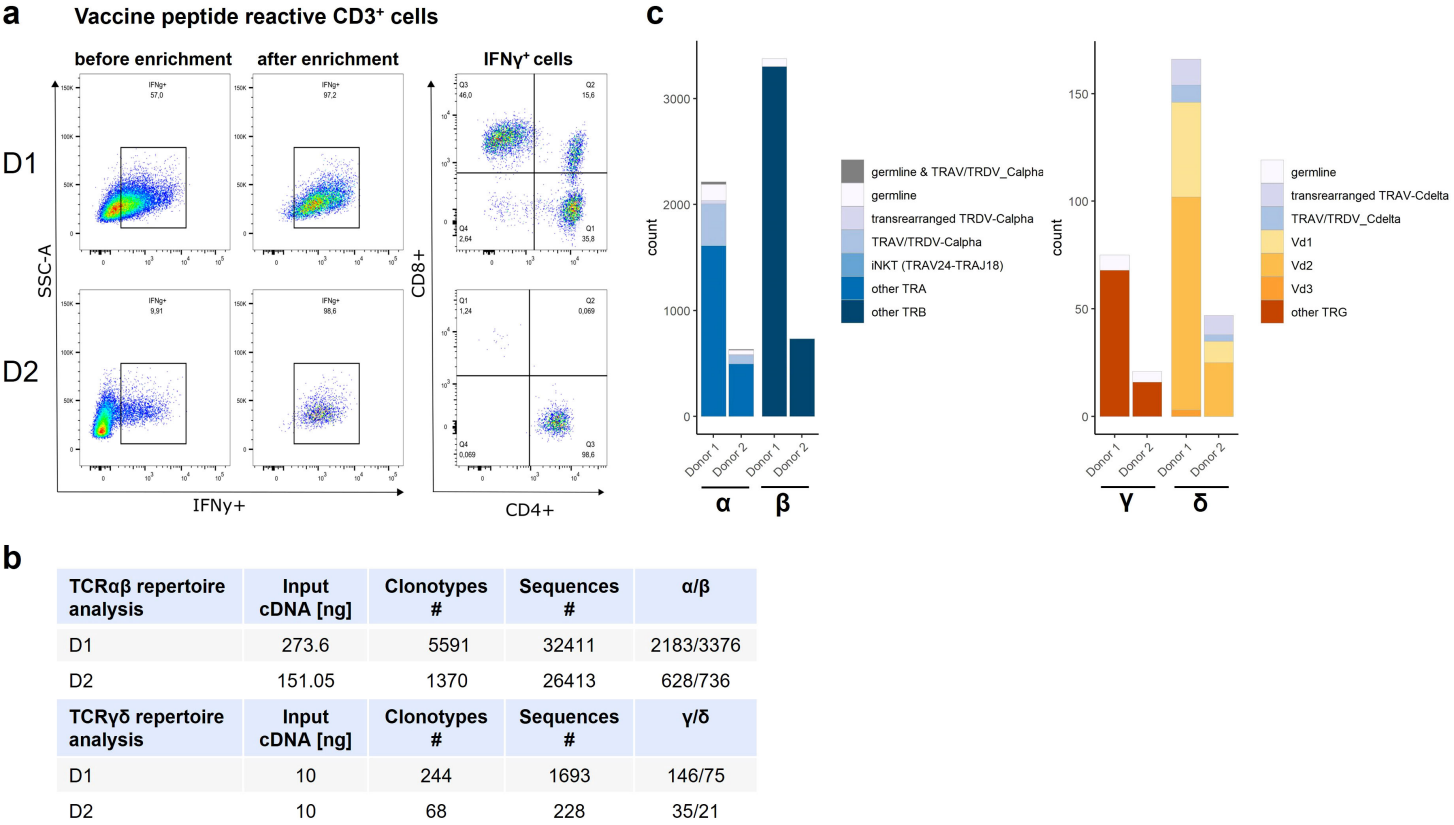
Coreceptor expression of Vaccine peptide reactive T cell pool and rearrangement type of α, β, γ, and δ clonotypes. **(a)** T cells responding to 16mer vaccine peptide with IFNγ secretion included CD8+ and CD4+ T cells in D1 (analyzed 4 weeks post inoculation) and were almost exclusively CD4+ in D2 (five months after inoculation). **(b)** left: TCRα and TCRβ clonotypes for donor 1 and donor 2 and TCRγ and TCRδ clonotypes (right). Details of the sequences are given, such as germline configuration, transrearrangements, iNKT clonotype. **(c)** comprehensive visualization of retrieved clonotypes from two separate sequencing runs for α, β, γ, and δ TCR clonotypes, color-coded and grouped by their compositional characteristics. The listed categories are defined as follows: germline category is defined as clones without N-Nucleotide insertions at the V(D)J junctions, germline & TRAV/TRDV_Calpha contains clones that are germline encoded and their α-segment is annotated as both TRAV and TRDV. Transrearranged TRDV-Calpha category comprises all clones in which the TCRα chain is generated by a rearrangement of TRDV1, TRDV2 or TRDV2 to a Cα segment. TRAV/TRDV-Cα includes all clones in which the TCR α-segment V-Region originated from a segment annotated as both TRAV and TRDV and that are linked to a Cα constant region. iNKT clones are characterized by a TCR α chain generated through TRAV24-TRAJ18 rearrangement, “other” categories constitute all remaining clones that don’t fall in any of the above-mentioned categories. The following definitions specify the distinct γδ T-cell clone categories: the transrearranged TRAV-Cδ category includes clones characterized by the recombination of TRA V-segment with a Cδ constant region. TRAV/TRDV_Cδ encompasses clones with a TRDV segment that is classified simultaneously as TRAV and TRDV and are joined to a Cδ constant region. Vδ1, Vδ2, Vδ3 categories comprise all clones that exhibit the respective TRDV rearrangement (Vδ1, Vδ2 and Vδ3, respectively).

Peptide-responsive T cells isolated from enrichment culture via IFNγ-secretion were mostly CD4^+^ in D2 (96%) whereas in D1, vaccinated only 4 weeks prior, also included CD8^+^ T cells (CD8: 46%, CD4: 35%) (**Figure 2a**). Peptide-responsive T cell`s RNA was extracted and subjected to NGS-based TCR-bulk sequencing (**Figure 1**).

TCR-profiling (**Figure 2b**) identified 32.411 sequences belonging to 5591 clonotypes with 2183 TCRα and 3376 TCRβ clonotypes for D1 and 26. 413 sequences belonging to 1370 clonotypes with 628 TCRα and 736 TCRβ clonotypes for D2 (**Figure 2b**, left). Separate TCRy/δ repertoire analysis (input: 10ng same cDNA) identified 1693 sequences/244 clonotypes (146 TCRδ and 75 TCRγ clonotypes) for D1 and 228 sequences/68 clonotypes (35 TCRδ/21 TCRγ clones) in D2 (**Figure 2b**, right), showing that also innate T-cell compartment responded to the vaccine. The compositional characteristics of the retrieved clonotypes for α, β, γ, and δ TCR chains are displayed in **Figure 2c**.

### CDR3-length profiles reveal focusing of the repertoire

**Figure 3:**
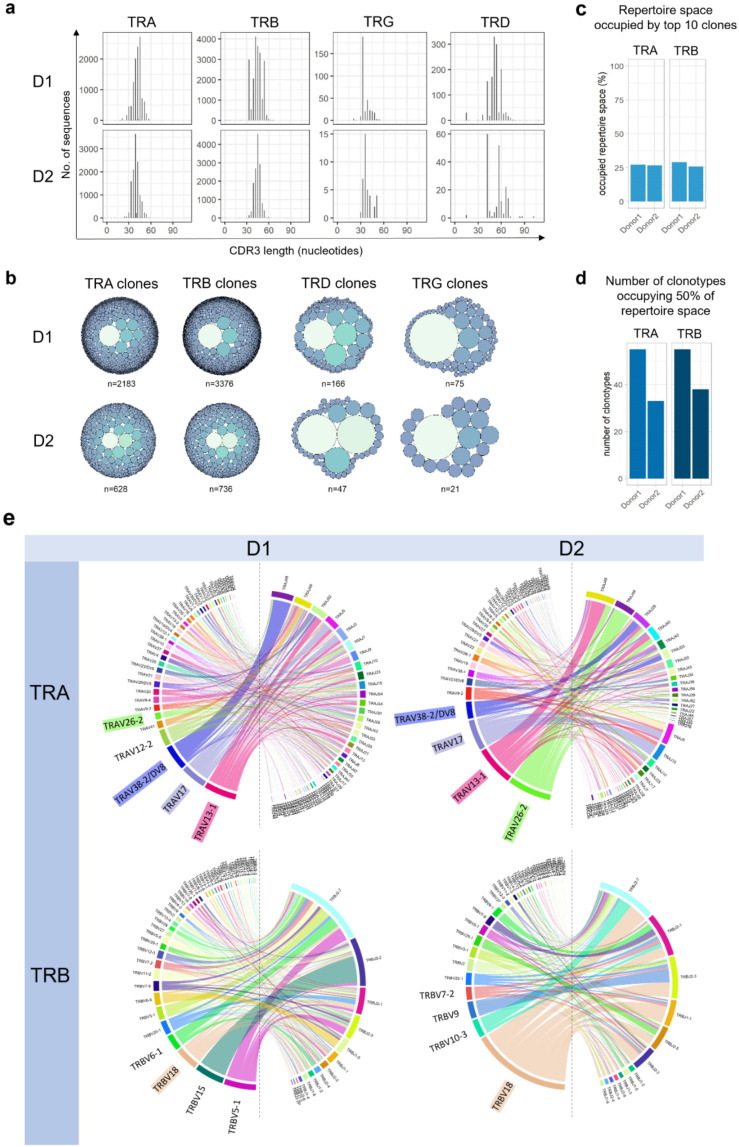
Physiochemical features of overlap repertoire. **(a)** The CDR3 length profiles of the α, β, γ, and δ clonotypes of both donors show a similarity of δ to the α and β clonotypes, as they all have a significant number of clonotypes expressing different CDR3 length transcripts, clearly breaking the Gaussian pattern. **(b)** Clonal distribution in α, β, γ, and δ TCR chain clonotypes displayed as circular tree blots. **(c)** The top ten α and top ten β clones in donors 1 and 2, respectively, as a percentage of the total α and β repertoire. **(d)** Number of clonotypes representing 50% of the TRA and TRB immune responses in donors 1 and 2, respectively. **(e)** Chord diagrams showing V(D)J usage reveal the same preferential V segment usage in both donors (highlighted in color).

CDR3-length profiles differed from normal Gaussian in both donors in all TCR-chains (**Figure 3a**). γ-clonotypes were focusing to a specific CDR3 length, δ-clonotypes showed some deviation, similar to CDR3-α/β clonotypes.

### Visualization of clonal distribution highlights substantial clonal expansions

Circular tree plots show clonal distributions (**Figure 3b**). The TOP10 TCRα-clones represented 27.2 and 26.7% of α-sequences in D1 and D2 (**Figure 3c**), the TOP10 TCRß-clones represented 29 and 25.8% of the β-sequences in D1 and D2, respectively (**Figure 3c**). 50% of the TRA-sequences consisted of 55 (D1) and 33 (D2) clonotypes, 50% of the TRB-sequences consisted of 55 (D1) and 38 clones (D2) respectively (**Figure 3d**).

### Preferred V-segment usage in TRAV and TRBV-clonotypes yet highly diverse CDR3 regions

Chord diagrams identified a shared preference for TRAV-segments 13-1, 17, 26-2, and 38-2/DV8 in both donors and a preference for TRBV 5-1, 15, 18 segments in D1 and TRBV-18, 10-3, 9 in D2 (**Figure 3e**).

A recent landmark study demonstrates that biochemical features are linked to the selection of V-genes (62) with germline-encoded CDR**1** region contacting the influenza A virus peptide thereby influencing (for spatial reasons) V-segment usage in TCRα and -β chains. For the same TCRs, it is shown that the germline-encoded parts of the CDR**3** region of the TCRα chain do not contact the target peptide, but only one or a few N-nucleotide-encoded amino acids which, very importantly, are identical or charge-conserved in TCRs of the same specificity.

Successive studies showed the same result (63, 64). Our results support this as a generally valid modus operandi too, assuming that six TRBV-segments used by approximately half of responding αβT cells in both donors, with three segments being identical, is a reasonably strong argument. Consistent with hydrophobic target recognition, the CDR1 regions of the preferentially used TRAV-segments (TRAV13-1*01, TRAV17*01, TRAV26-2*01 in both, plus TRAV38-2/DV8 in D1), show up to five aliphatic/hydrophobic amino acids (aa), and CDR1 of TRDV1 four respectively (**Table 2**).

**Table 2:** CDR1 and CDR2 Regions of the most frequently used TRAV segments and the Vδ1 segment.

Germline_v_call	CDR1	CDR2
TRAV13-1*01	DS**A*SN*Y**	IR***SN***VGE
TRAV17*01	TS**I**NN	IR***SN***ERET
TRAV26-2*01	T**I**S**G**TD**Y**	GLT***SN***
TRAV38-2/DV8	**PV**R**VII**	KKLI***SN***R
TRDV1	TS**WW**S**YY**	QG***S***

Motif SN (blue, italic) is present in the CDR2 regions in the most common TRAV segments and additionally in CDR1 of TRAV 13. Vδ1 segment contains 4 highly aliphatic aa in its CDR1 and S (blue and bold) in its CDR2 segment. Aliphatic amino acids are given in red and bold.

The CDR2 regions of preferred TRAV segments share the motif SN, consistent with germline-encoded CDR2-binding to conserved regions of the MHC-anchor molecule. The CDR2-loop of the Vδ1-segments is described to interact with lipid-presenting CD1 molecules (65) (**Table 2**).

In contrast, the corresponding CDR3 regions of these most expanded clonotypes are highly diverse, lack a conserved motif (**Table 3**), have few N-nucleotides (**Table 3**, underlined) predominantly encoding aliphatic/hydrophobic aa-insertions.

**Table 3:** Identical V segments usage in D1 and D2 by the most expanded TRAV and TRBV clonotypes (n≥1000).

Most highly expanded clonotypes carrying the most commonly used TRAV segments (left) and TRB segment (right)								
TRA				TRB				
Donor	CDR3	V	J	Donor	CDR3	V	D	J
D1	CATD**AR**T**G**RRALTF	TRAV17	TRAJ5	D1	CASS**LR**Q**GV**NTEAFF	TRBV18	TRBD1	TRBJ1-1
	CAA**V**N**AGG**TSYGKLTF	TRAV13-1	TRAJ52		CASS**P**S**PG**TDTQYF	TRBV18		TRBJ2-3
	CATD**RKG**SSASKIIF	TRAV17	TRAJ3		CASS**P**S**K**Q**GL**SPLHF	TRBV18	TRBD1	TRBJ1-6
	CAAS**PRIII**Q**GA**QKLVF	TRAV13-1	TRAJ54		CASS**P**T**GGAM**NEQFF	TRBV18	TRBD1	TRBJ2-1
	CAA**RGV**YNFNKFYF	TRAV13-1	TRAJ21		CASS**PKVG**T**KG**SNEQFF	TRBV18	TRBD2	TRBJ2-1
	CAA**RA**DKLIF	TRAV13-1	TRAJ34		CASS**PRL**T**G**S**G**NSPLHF	TRBV18	TRBD1	TRBJ1-6
	CAT**AT**NS**GG**YQKVTF	TRAV17	TRAJ13		CASS**PG**T**GA**YNEQFF	TRBV18	TRBD1	TRBJ2-1
	CAAS**IGI**YNFNKFYF	TRAV13-1	TRAJ21		CASS**PR**SS**GGA**NTGELFF	TRBV18	TRBD1	TRBJ2-2
	CAT**GLLI**Q**GA**QKLVF	TRAV17	TRAJ54		CASS**PRLAGGL**SSYNVQFF	TRBV18	TRBD2	TRBJ2-1
	CAS**RGA**SKIIF	TRAV13-1	TRAJ3		CASS**PRAG**T**G**SPGELFF	TRBV18	TRBD1	TRBJ2-2
	CAA**L**SYNQGGKLIF	TRAV13-1	TRAJ23		CASS**PG**STTGELFF	TRBV18		TRBJ2-2
	CATS**GG**NNRLAF	TRAV17	TRAJ7		CASS**PA**S**GRAGA**NVLTF	TRBV18	TRBD2	TRBJ2-6
	CAQSTYNQGGKLIF	TRAV13-1	TRAJ23		CASS**PAPG**QTNEQYF	TRBV18	TRBD1	TRBJ2-7
	CA**PR**N**G**S**GG**TSYGKLTF	TRAV13-1	TRAJ52		CASS**PGAR**NSPLHF	TRBV18	TRBD1	TRBJ1-6
	CA**F**T**G**NQFYF	TRAV17	TRAJ49		CASS**IK**T**G**NP**LV**NNEQFF	TRBV18	TRBD1	TRBJ2-1
D2	CAAS**ILGG**NQFYF	TRAV13-1	TRAJ49	D2	CASS**R**SP**G**Q**A**DTQYF	TRBV18		TRBJ2-3
	CIL**V**TS**G**TYKYIF	TRAV26-2	TRAJ40		CASS**PG**T**G**NTEAFF	TRBV18	TRBD1	TRBJ1-1
	CIL**RGG**NTGNQFYF	TRAV26-2	TRAJ49		CASS**I**SS**G**TSTDTQYF	TRBV18	TRBD2	TRBJ2-3
	CIL**RVWGF**GNEKLTF	TRAV26-2	TRAJ48		CASS**L**NY**VRG**QETQYF	TRBV18	TRBD1	TRBJ2-5
	CAAS**H**S**G**NTPLVF	TRAV13-1	TRAJ29		CASS**A**ST**GV**NEQFF	TRBV18	TRBD1	TRBJ2-1
	CILLS**G**NTPLVF	TRAV26-2	TRAJ29		CASS**PLG**TS**GR**NQETQYF	TRBV18	TRBD2	TRBJ2-5
	CAASTS**G**TYKYIF	TRAV13-1	TRAJ40		CASS**RG**T**GAL**NVLTF	TRBV18	TRBD1	TRBJ2-6
	CAASSN**G**GNQFYF	TRAV13-1	TRAJ49		CASS**PVKVAL**SGNTIYF	TRBV18		TRBJ1-3
	CIL**KGG**N**A**GNMLTF	TRAV26-2	TRAJ39		CASS**PV**P**IGVG**TDTQYF	TRBV18		TRBJ2-3
	CIL**R**D**WGG**EKLTF	TRAV26-2	TRAJ48		CASS**P**S**IV**S**GH**EQYF	TRBV18		TRBJ2-7
	CIL**R**D**VA**NFGNEKLTF	TRAV26-2	TRAJ48		CASS**PGLVG**SEQFF	TRBV18		TRBJ2-1
	CAASTS**G**TYKYIF	TRAV13-1	TRAJ40		CASS**K**S**PP**NTGELFF	TRBV18		TRBJ2-2
	CAAS**KPG**NNRKLI	TRAV13-1	TRAJ38		CASS**P**S**KIG**T**G**AYEQYF	TRBV18	TRBD1	TRBJ2-7
	CAA**RG**SDNRLAF	TRAV13-1	TRAJ7		CASS**RG**TNNSPLHF	TRBV18	TRBD2	TRBJ1-6
	CAAS**F**Y**GG**SQGNLIF	TRAV13-1	TRAJ42		CASS**RGR**QFEQYF	TRBV18		TRBJ2-7

TRAV segments (left) and TRB segment (right). Aliphatic (hydrophobic) amino acids given in green and bold, basic amino acids in red and bold. N nucleotide encoded aa are underlined.

### Public and superpublic TCRs emerge from repertoire alignment

Alignment of repertoires according to identical sequences provides information on the proportion of so-called public TCRs.

**Figure 4:**
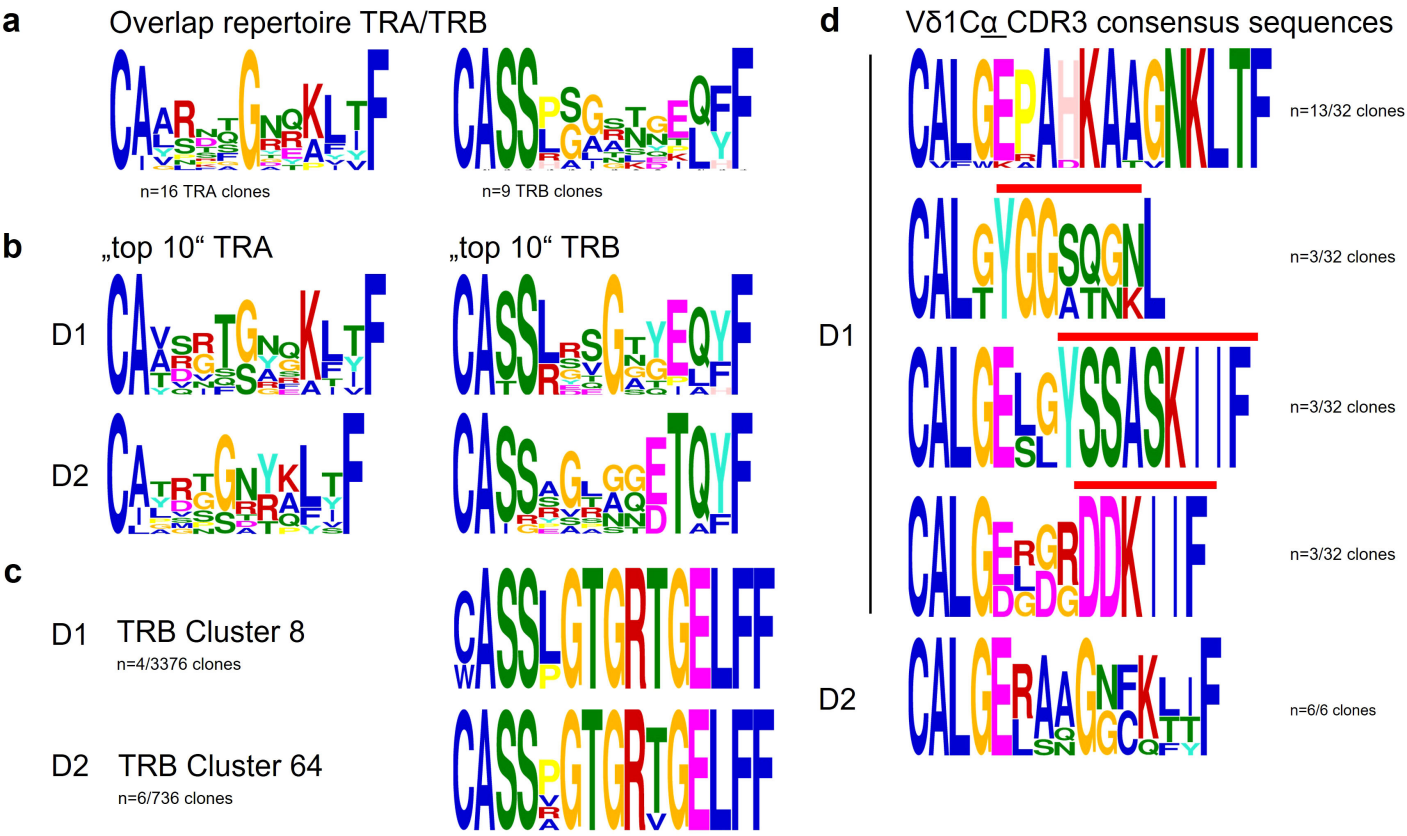
Consensus sequences. **(a)** CDR3 α and β chain LOGO BLOTS of the overlap repertoire show the conservation of a glycine (G) at position 111 in the TRA clonotypes, in contrast TRBV sequences lack a conserved motif/position. **(b)** Top 10 TRAV and TRBV clonotypes (immunodominant) show no sequence/motif conservation but are highly individual. **(c)** D1 and D2 clonotypes of most frequent TRB CDR3 length are private (not shown), only one tiny cluster is shared between the donors (D1 n=4, D2 n=6 clonotypes). **(d)** Vδ1Cα clonotypes grouped in 4 (D1), respectively 1 cluster (D2) share motifs with Covid-19 reactive TRAV αβ T cell clonotypes (YGGSQGN, YSSASKIIF, DDKIIF) 62,63,64.

The small overlap of repertoire between the donors included 15 TRAV and 9 TRBV-clonotypes, two clonotypes combining a Vα with a Cδ-segment (TRAV12/TRAJ20 and TRAV12/TRAJ44 with Cδ (LPVSF), clonotype TRAV23/DV6/DD2-DJα- Cα (VLPALLSQTQLGSFPPLRP) and the most frequent γδ-T-cell clone HCSHFLDPYSALCV (**Table 4**). Clonotypes were either derived from rearrangements involving same or different V(D)J segments (convergent CDR3 formation). Logoplots of the overlap repertoire between both donors for TRAV and TRBV-clonotypes reveal a glycine (G) at position 111 in the TRAV clonotypes, whereas the TRBV sequences lack a conserved motif/position (**Figure 4a**).

**Table 4:** Overlap repertoire TRA, TRB, TRD Overlap sequences lack or show only few N nucleotides.

Locus	Rearrange-ment	CDR3 V	…N…	J	V-segment	D segment	J segment	Np1_length	Np2_length	Donor	# of clones	CDR3 sequence	Public
TRA	VαCα	**CAG**	**RRR**	**QGAQKLVF**	TRAV35*01		TRAJ54*01	8		D1+D2	1 (D1)/2 (D2)	Ident.	+
	VαCα	**CAYRS**	**F**	**SGNTPLVF**	TRAV38-2/DV8*01		TRAJ29*01	5 (D1)/3 (D2		D1+D2	1 (D1)/1 (D2)	Con.	+
	VαCα	**CAYRS**		**YGGSQGNLIF**	TRAV38-2/DV8*01		TRAJ42*01	2 (D1)/0 (D2)		D1+D2	1 (D1)/1 (D2)	Con.	+
	VαCα	**CILR**	**V**	**NFGNEKLTF**	TRAV26-2*01		TRAJ48*01	2 (D1)/3 (D2)		D1+D2	1 (D1)/1 (D2)	Con.	+
	VαCα	**CILR**	**AP**	**FGNEKLTF**	TRAV26-2*01		TRAJ48*01	4 (D1+D2)		D1+D2	1 (D1)/1 (D2)	Ident.	+
	VαCα	**CA**	**V**	**TGNQFYF**	TRAV13-1*01		TRAJ49*01	5 (D1)		D1	1 (D1)	Con.	+
		**CAV**		**TGNQFYF**	TRAV21*01		TRAJ49*01	0 (D2)		D2	1 (D2)		+
	VαCα	**CAA**	**R**	**DTGRRALTF**	TRAV13-1*01		TRAJ5*01	3 (D1)		D1	1 (D1)	Con.	+
					TRAV13-1*02		TRAJ5*01	0 (D1+D2)		D1+D2	1 (D1)/1 (D2)	Con.	+
	VαCα	**CAAS**	**KA**	**GRRALTF**	TRAV13-1*01		TRAJ5*01	3 (D1)		D1	1 (D1)	Ident.	+
					TRAV13-1*02		TRAJ5*01	3 (D2)		D2	1 (D2)		+
	VαCα	**CAAS**		**TSGTYKYIF**	TRAV13-1*01		TRAJ40*01	0 (D1)		D1	1 (D1)	Con.	+
					TRAV13-1*02		TRAJ40*01	0/2 (D2)		D2	6 (D2)	Con.	+
	VαCα	**CAAS**		**NQGGKLIF**	TRAV13-1*01		TRAJ23*01	3 (D1)		D1	1 (D1)	Ident.	+
					TRAV13-1*02		TRAJ23*01	4 (D2)		D2	1 (D2)		+
	VαCα	**CA**	**L**	**RDDKIIF**	TRAV21*01		TRAJ30*01	0 (D1)		D1	1 (D1)	Con.	+
		**CAL**		**RDDKIIF**	TRAV19*01		TRAJ30*01	0 (D2)		D2	1 (D2)		+
	VαCα	**CAV**		**NSGGYQKVTF**	TRAV2*01		TRAJ13*02	1 (D1)		D1	2 (D1)	Con.	+
		**CAVN**		**SGGYQKVTF**	TRAV12-2*01		TRAJ13*02	0 (D2)		D2	1 (D2)	Con.	+
	VαCα	**CAA**	**P**	**NTGNQFYF**	TRAV29/DV5*04		TRAJ49*01	1 (D1)		D1	1 (D1)	Con.	+
					TRAV29/DV5*01		TRAJ49*01	3 (D2)		D2	1 (D2)		+
	VαCα	**C**	**D**	**NNNDMRF**	TRAV16*01		TRAJ43*01	0 (D1+D2)		D1+D2	1 (D1+D2)	Ident.	+
	VαCα	**CAA**	**L**	**DTGRRALTF**	TRAV13-1*01		TRAJ5*01	1 (D1+D2)		D1+D2	1 (D1+D2)	Con.	+
	VαJδCα	**VLPAL**	**L**	**SQTQLGSFPPLRP**	TRAV23/DV6*01	TRDD2*01	TRDJ4*01	**48** (D1+D2)	**8** (D1+D2)	D1+D2	1 (D1+D2)	Ident.	**no**
TRB	VβCβ	**CASSHGTSGR**	**L**	**GELFF**	TRBV3-1*01	TRBD2*02	TRBJ2-2*01	1	3 (D1)/2 (D2)	D1+D2	1 (D1)/1 (D2)	Con.	+
	VβCβ	**CASSP**	**GIS**	**SYEQYF**	TRBV18*01		TRBJ2-7*01	8 (D1)/9 (D2)		D1+D2	1 (D1)/1 (D2)	Con.	+
	VβCβ	**CASSL**	**SLS**	**TDTQYF**	TRBV11-2*03		TRBJ2-3*01	9 (D1)		D1	1 (D1)	Con.	+
					TRBV11-2*01		TRBJ2-3*01	7/6 (D2)		D2	2 (D2)	Con.	+
	VβCβ	**CASS**	**PSNG**	**AKNIQYF**	TRBV7-2*01		TRBJ2-4*01	11 (D1)/12(D2)		D1+D2	1 (D1)/1 (D2)	Con.	+
	VβCβ	**CASSPGTG**	**R**	**TGELFF**	TRBV18*01,	TRBD1*01	TRBJ2-2*01	1 (D1)/0/1 (D2)	3(D1)/2/3 (D2)	D1+D2	1 (D1)/2 (D2)	Con.	+
	VβCβ	**CASSP**	**SP**	**ANTGELFF**	TRBV18*01		TRBJ2-2*01	8 (D1+D2)		D1+D2	1 (D1)/1 (D2)	Con.	+
	VβCβ	**CASSLGG**		**GNQPQHF**	TRBV12-3*01	TRBD1*01	TRBJ1-5*01	0 (D1)/8 (D2)	1 (D1+D2)	D1+D2	1 (D1)/1 (D2)	Con.	+
	VβCβ	**CASRAG**	**A**	**NNEQFF**	TRBV7-9*01	TRBD2*01	TRBJ2-1*01	1 (D1)	3 (D2)	D1	1 (D1)	Con.	+
					TRBV7-9*03	TRBD2*01	TRBJ2-1*01	1 (D2)	5 (D2)	D2	6 (D2)	Con.	+
	VβCβ	**CASSL**	**S**	**ATNEKLF**	TRBV12-3*01		TRBJ1-4*01	5 (D1)/4 (D2)		D1+D2	1 (D1)/2 (D2)	Con.	+
TRD	VαCδ	**LPVSF**			TRAV12-1*01		TRAJ44*01	8 (D1+D2)		D1+D2	1 (D1+D2)	Ident.	+
							TRAJ20*01	8 (D1+D2)		D1+D2	1 (D1+D2)	Ident.	+
	VδCδ	**HCSHFLDPYSALCV**			TRAV36/DV7*01	TRDD2*01	TRDJ2*01	**58** (D1+D2)	**22** (D1+D2)	D1+D2	1 (D1	Ident.	**no**

CDR3 regions are displayed in bold. Aliphatic amino acids are given in green, basic amino acids in red.

Abbreviations in CDR3 sequence column: "ident." for identical sequences, "con." for convergent sequences. Convergent sequences show the same nucleotide sequence but are derived from rearrangments involving different VDJ segments. Screening for the public nature of a clonotype was performed using the iReceptor Scientific Gateway of the iReceptor platform (60).

### Overlap and adaptive clonotypes resemble neonatal TCRs

Recent studies have shown that the adaptive TCR repertoire consists of two ontogenetically and functionally distinct TCR types, which are regulated by variations in thymic production and terminal deoxynucleotidyl transferase (TDT) activity (66). Strikingly, the CDR3 sequences of the overlap repertoire and the most common vaccine-reactive αβ T clonotypes (**Table 5**) lack or have only few N nucleotides, assigning them to the neonatal TCRs derived from TDT-negative precursors, which persist throughout life, are highly shared between individuals and are reported to be disease-associated (66).

**Table 5:** TRA and TRB top 10 clonotypes in D1 and D2.

Top 10 Clonotypes										
TRA					TRB					
Donor	CDR3	V	J	Public +/-	Donor	CDR3	V	D	J	Public +/-
D1	CAY**R**S**SRFG**NEKLTF	TRAV38-2/DV8*01	TRAJ48*01	+	D1	CATS**RVG**Q**GRLR**TGELFF	TRBV15*02	TRBD1*01	TRBJ2-2*01	-
	CATD**AR**T**GRR**ALTF	TRAV17*01	TRAJ5*01	+		CASS**RSSGIP**SYEQYF	TRBV5-1*01	TRBD2*02	TRBJ2-7*01	-
	CATD**RKG**SSASKIIF	TRAV17*01	TRAJ3*01	-		CASSYS**G**SNQPQHF	TRBV6-5*01	TRBD2*01	TRBJ1-5*01	+
	CAA**V**N**AGG**TSYGKLTF	TRAV13-1*02	TRAJ52*01	+		CASSE**SFM**AYEQYF	TRBV6-1*01		TRBJ2-7*01	-
	CAVS**V**T**GG**FKTIF	TRAV8-4*01	TRAJ9*01	+		CASSS**A**YEQYF	TRBV7-2*02		TRBJ2-7*01	+
	CAV**RLS**NTGNQFYF	TRAV41*01	TRAJ49*01	+		CS**GVGG**YEQYF	TRBV29-1*01	TRBD1*01	TRBJ2-7*01	+
	CAAS**MR**TSYGKLTF	TRAV23/DV6*01	TRAJ52*01	+		CASS**LR**Q**G**VNTEAFF	TRBV18*01	TRBD1*01	TRBJ1-1*01	+
	CAVQ**DTQG**TGNQFYF	TRAV20*02	TRAJ49*01	-		CASSD**SGGG**IQYF	TRBV6-1*01	TRBD2*01	TRBJ2-4*01	+
	CAAS**PRIII**QGAQKLVF	TRAV13-1*02	TRAJ54*01	-		CASS**LG**T**GR**TGELFF	TRBV5-5*01	TRBD1*01	TRBJ2-2*01	+
	CGGNNRLAF	TRAV12-2*01	TRAJ7*01	+		CAS**R**E**GA**TYEQYF	TRBV7-9*01	TRBD1*01	TRBJ2-7*01	+
D2	CATD**PS**AALIF	TRAV17*01	TRAJ15*01	-	D2	CASS**VRALA**GPDTQYF	TRBV9*01	TRBD2*02	TRBJ2-3*01	+
	CAAS**ILGG**NQFYF	TRAV13-1*02	TRAJ49*01	+		CAISE**GLAG**VYEQYF	TRBV10-3*02	TRBD2*02	TRBJ2-7*01	+
	CAY**R**S**Q**S**G**NTPLVF	TRAV38-2/DV8*01	TRAJ29*01	+		CASS**RSPGQ**ADTQYF	TRBV18*01		TRBJ2-3*01	+
	C**LGM**DT**GRR**ALTF	TRAV22*01	TRAJ5*01	+		CASS**PG**T**G**NTEAFF	TRBV18*01	TRBD1*01	TRBJ1-1*01	+
	CAY**RGRGGG**SNYKLTF	TRAV38-2/DV8*01	TRAJ53*01	+		CS**GWLAGSGG**ETQYF	TRBV29-1*01	TRBD2*02	TRBJ2-5*01	-
	CIL**V**TS**G**TYKYIF	TRAV26-2*01	TRAJ40*01	+		CAS**RAGA**NNEQFF	TRBV7-9*03	TRBD2*01	TRBJ2-1*01	+
	CA**PR**NDYKLSF	TRAV17*01	TRAJ20*01	+		CASS**I**SS**GT**STDTQYF	TRBV18*01	TRBD2*02	TRBJ2-3*01	+
	CATD**RGGGG**NKLTF	TRAV17*01	TRAJ10*01	+		CASS**LNYVRGQ**ETQYF	TRBV18*01	TRBD1*01	TRBJ2-5*01	-
	CAT**GH**T**GRR**ALTF	TRAV17*01	TRAJ5*01	+		CASS**AGLAG**EQYF	TRBV2*01	TRBD2*01	TRBJ2-7*01	+
	CIL**RGG**NT**G**NQFYF	TRAV26-2*01	TRAJ49*01	+		CASS**PLG**TS**GR**NQETQYF	TRBV18*01	TRBD2*02	TRBJ2-5*01	-

N-Nucleotide encoded amino acids are underlined and bold, alphatic amino acids are green and bold, basic amino acids are red and bold. Screening for the public nature of a clonotype was performed using the iReceptor Scientific Gateway of the iReceptor platform (60).

In line with these findings, we also found germline-encoded TCRs making up 8.11% (177/2183) and 7.8% (49/2682) of TRAV-clonotypes in D1/D2, and 2.13 and 0.68% of TRBV-clonotypes in D1/D2 respectively.

In contrast to the TDT-negative precursors, TDT-dependent TCRs with distinct structural features and less shared among subjects (66) was unexpectedly represented by a γδ T-cell clone (HCSHFLDPYSALCV **Table 3**, TRD bottom line) and a VαJδCα hybrid clone (VLPALLSQTQLGSFPPLRP, **Table 4**) in the overlap repertoire.

Both sequences showing atypical features with 58 and 48 Np1 inserts between V- and D-region and 22 and 8 Np2 inserts between D- and J-region respectively (**Table 4**).

### Search for motifs in αβ-clonotypes

#### TOP10 SARS-CoV2nuc-reactive clonotypes are completely diverse and superpublic

To analyze the entirety of αβ-clonotypes in a biologically meaningful way (67–69) TOP10 clonotypes, which can be considered immunodominant due to their prevalence, and the most common V(D)J-rearrangements were examined for conserved positions/sequence similarities in their CDR3 region. TOP10 clonotypes (**Table 5**) were not similar to each other nor were they identified in the TCR-repertoire of the other donor. They also did not show a conserved motif (**Figure 4b**), but in both doners the majority of TOP10 clonotypes was superpublic (present in a large proportion of a studied population) and annotated in public databases as part of Covid-19 immune responses (**Table 5**).

Also, most frequent V(D)J-rearrangements had no conserved motif in their CDR3 regions (**Table 3**).

### Clonotypes with most frequent CDR3-length are exclusively private

Clonal expansions can significantly bias CDR3-length distribution. Yet, GLIPH2 algorithm analysis of most frequent CDR3 length clonotypes (D1: 45 nucleotides; 742 clonotypes, D2–42 nucleotides: 135 clonotypes) identified very few and small clusters. A tiny TRB-cluster with similar motif (**Figure 4c**) was present in both donors, yet the vast majority of clonotype-clusters was completely private in both donors (not shown).

A private clonotype-cluster refers to a group of immune cells (typically T or B lymphocytes) that share highly similar or identical antigen receptor sequences, but are unique to a single individual, sample, or patient, rather than being found across multiple individuals. This distinguishes them from public clonotypes, which are shared between different individuals and often reflect common immunological responses to well-conserved antigens.

### Immunodominant clonotypes show N-nucleotide encoded hydrophobicity in their CDR3 region

To identify consensus criteria of vaccine-reactive clonotypes we followed Ritmahan et al (63) who showed that factors that determine whether a response becomes immunodominant (ID) per donor is that their CDR3 regions distinctively show hydrophobic aa residues compared to the subdominant (SD) responses. Since the common V-J combinations are shared between ID and subdominant SD responses, the authors assume that the biased V-J recombination events are restricted by epitope specificity; immunodominance however resulting from detectable distinctive amino acid enrichments i.e. hydrophobic amino acids in D-N encoded CDR3 regions. We found matching results.

Removing the conserved V- and J-regions of the CDR3 sequences at the terminal ends, leaves a “non-VJ”-region as core sequence encoded by randomly inserted nucleotides. For TRAV-sequences between V and J, for TRB-segments encoded by D-segment and N-nucleotide insertions. Immunodominant clonotypes, i.e. TOP10 TRAV/TRBV-clonotypes (**Table 5**) and clonotypes incorporating preferred V-(D)-J rearrangements (**Table 3**) coherently show hydrophobic (aliphatic) amino acids (listed for increasing hydrophobicity: G,P,A,V,I,L,M,F,W) in their N-nucleotide encoded CDR3 regions (red).

We find germline-encoded CDR3 clonotypes combine aliphatic with basic amino acids, immunodominant CDR3 clonotypes combining aliphatic with basic or neutral polar uncharged amino acids (**Table 3**), while rarer clonotypes combine aliphatic amino acids with electrically charged amino acids (not shown). Importantly, 7/11 TdT-independent “neonatal” TCR clonotypes, which have N-nucleotides, if any, have aliphatic/hydrophobic amino acid “insertions” (**Table 4**).

### Vδ1-Cα clonotypes link αβTCR diversity with innate-like recognition

Classically TCR-chains are encoded by genes formed by elements belonging to the same locus. However, trans-rearrangements between V(D)JC elements belonging to different TCR-chain loci have been described (64, 70–73), most of them fusing Vγ and (D)Jβ elements, which are translated into functional antigen-receptor chains that couple with TCRα-chains (74). γβ-clonotypes were absent in our study. However, the δ-locus (8 variable segments) is interspersed within the α-locus, and each of the Vδ-genes can productively rearrange to a Jα-segment, producing functional Vδ-Jα-Cα chains that can pair with a TCRβ chain and form functional αβ-TCRs (74, 75). Vδ1, the most upstream Vδ-gene-segment, can rearrange to almost all Jα-gene-segments (76), which we found confirmed in the Vδ1-Cα sequences of D1 and D2 (42(1.4%) and 9(0.9%) of TRAV-clonotypes respectively), carrying J-segments derived from the entire Jα-gene locus (Jα3 to Jα48 in D1, Jα9 to Jα49 in D2). No Vδ2-Cα, Vδ3-Cα, or Vγ-Cβ trans-rearrangements were identified (77).

Four Vδ1-Cα clonotype-clusters in D1 share motifs with public Covid-19 reactive TRAV αβ-T-cell clonotypes (**Figure 4d**): YGGSQGN is the major motif in Covid 19-reactive TRAV-clonotypes of CD4^+^ αβ-T cells in reconvalescent individuals (78), YSSASKIIF characterizes the TRAV19-Jα3 rearrangement in a Covid-specific CD8^+^ αβ-T cell clone (79) and motif DDKIIF is part of a public αβ-TCR specific for the highly hydrophobic Covid-derived spike peptide YLQPRTFLL (80).

Six Vδ1-Cα sequences, detected in D2, formed two clusters (three and two sequences, with almost identical sequences) (**Supplementary Table 1**).

### Vδ1–Cα clonotypes represent a distinct subset integrated within the αβ TCR repertoire

**Figure 5:**
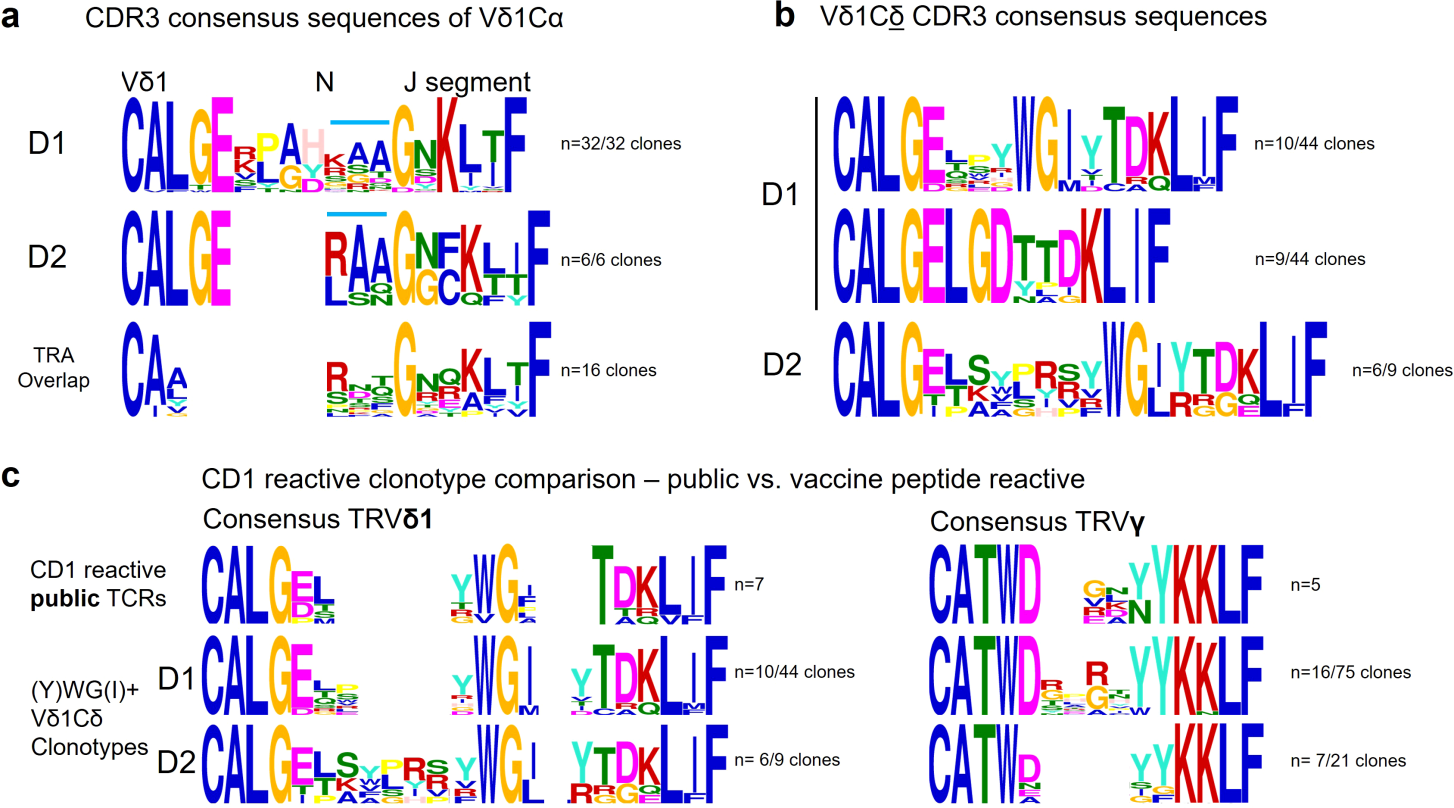
Search for motif conservation. In **(a)** Comparison of CDR3 consensus sequences of Vδ1Cα with TRAV overlap **(b)** Comparison of Vδ1Cδ CDR3 consensus sequences in D1/D2 **(c)** Homology of public CD1 reactive γδ TCRs; Left: comparison public CD1 reactive TCRδ clonotypes with vaccine peptide reactive TRDVδ1 chain clonotypes containing (Y)WG(I), Right: TCRγ chain clonotypes manually clustered compared to consensus TCRγ CDR3 sequences of public CD1-restricted TCRγ clonotypes.

CDR3 consensus of Vδ1-Cα-clonotypes show an N-nucleotide encoded 3-aa motif between germline-derived 5` and 3` ends with charge conservation for hydrophobicity (blue horizontal line) (**Figure 5a**). The CDR3 consensus generated from the α-clonotypes of the overlap repertoire (third line) shows the basic but lacks the aliphatic (AA)-residues.

Vδ1Cα-αβTCRs via their Vδ1-segment are preprogrammed in their CDR1/2 binding specificity for CD1 and MHC. They may act as pattern recognition receptors (PRR) for MHC molecules that are upregulated under inflammatory conditions, thus may represent a unique aspect of epithelial barrier immunity, combining innate biology with adaptive receptors.

The unexpected identification of VδCα-hybrid clonotypes led us to search for VδCδ and VγCγ clonotypes in the vaccine-reactive T-cell pools.

### Vaccine-reactive γδ-clonotypes

γδ-TCRs can recognize polypeptides that are soluble or membrane-anchored and cross-linked to MHC or MHC-like molecules (81) including CD1b, CD1c, CD1d and MHC class I-related protein 1 (MR1), recognizing antigen, antigen presenting molecule or both either via germline-encoded regions of the receptor, reminiscent of PRRs, or adaptive antigen binding via the CDRs (82–85). In doing so, γδ-T cells are not restricted to the contact mode of αβ-TCRs but can approach their ligands from many directions (86).

### Most Vγ-clonotypes are Vγ9^negative^ and homologous to public CD1-restricted TCRγ-clonotypes

A putative CD1 presentation of the lipophilic vaccine peptide was investigated by aligning the sequences of public CD1-restricted TCR sequences with the γ- and δ-clonotypes of D1 and D2.

The public CD1-restricted TCRs DP10.7 (87), CO3 (88), BC14.1 (89) CDR3 TCRγ-chain region 5’-motif CATWD is germline-encoded, provided by TRGV-segments 1-8, the 3’-motif YYKKLF is derived from TRGJ1. (**Figure 5c**, **Table 6A**) (87–89).

**Table 6:** SARS-CoV-2-nuc peptide reactive γδ sequence repertoires include clonotypes that are highly homologous to TCRδ and γ chain clonotypes of public CD1 reactive γδ TCRs a) γ CDR3 clonotypes. b) CDR3 of (Y)WG(I)+ Vδ1 clonotypes.

A			
Public CD1 reactive TCRs	Vγ CDR3		
	CDR3 region	Vγ-segment	J-segment
DP10.7	CATWD EK YYKKLF	TRGV4*02	TRGJ1
AB 18.1	CATWD RNN KKLF	TRGV4*02	TRGJ2
C022	CATWD GVGA YYKKLF	TRGV2*01	TRGJ2
BC14.1	CATWD VLN YKKLF	TRGV4*02	TRGJ2
C03	CATWD GD YYKKLF	TRGV4*02	TRGJ2
Vγ clonotypes			
D1	CATWD YL YYKKLF	TRGV4*02	TRGJ1*02
	CATWD NPRN YKKLF	TRGV8*01	TRGJ1*02
	CATWD GLT KLF	TRGV4*02	TRGJ1*02
	CATWD GHGC YKKLF	TRGV4*02	TRGJ1*02
	CATWD GPN YYKKLF	TRGV4*02	TRGJ1*02
	CATWD THH YYKKLF	TRGV3*01	TRGJ1*02
	CATWD REAH YKKLF	TRGV3*01	TRGJ1*02
	CATWD TTRP YKKLF	TRGV8*01	TRGJ1*02
	CATWD SHT YYKNLF	TRGV8*01	TRGJ1*02
	CATWD A YKKLF	TRGV2*01	TRGJ1*02
	CATWD RD YYKKLF	TRGV3*01	TRGJ1*02
	CATWD GAG YKKLF	TRGV2*01	TRGJ1*02
	CATWD SRGVW YKKLF	TRGV5*01	TRGJ1*02
	CATWD RQRY YYKKLF	TRGV3*01	TRGJ1*02
	CATWD RPRI YYKKLF	TRGV3*01	TRGJ1*02
	CATWD CG YKKLF	TRGV4*02	TRGJ1*02
D2	CATWD G YYKKLF	TRGV2*03	TRGJ1*02
	CATWD GLT YYKKLF	TRGV4*02	TRGJ1*02
	CATWD GIG KKLF	TRGV5*01	TRGJ1*02
	CATW N YYKKLF	TRGV8*01	TRGJ1*02
	CATWE G YKKLF	TRGV4*02	TRGJ1*02
	CATW AM GYYKKLF	TRGV8*01	TRGJ1*02
	CATWD RQSF KKLF	TRGV3*01	TRGJ1*02

B				
Public CD1 reactive TCRs	Vδ1 CDR3			
	Vδ1 …N… D3 …N… J1/2	Vδ1-segment	D-segment	J-segment
DP10.7	CALGE PS **YWG** FPRT TRVIF	TRDV1*01	TRDD3*01	TRDJ1*01
AB 18.1	CALGD QIL **YWG**L SH TDKLIF	TRDV1*01	TRDD3*01	TRDJ1*01
Bai et al.	CALGD GIPL **YWGIL** TASY TDKLIF	TRDV1*01	TRDD3*01	TRDJ1*01
Bai et al.	CALGE LTV **G** AVH TDKLIF	TRDV1*01	TRDD3*01	TRDJ1*01
CO22	CALG PSYM **YWGI** ILD TDKLIF	TRDV1*01	TRDD3*01	TRDJ1*01
BC14.1	CALGE RTG **WGF** APLV TAQLFF	TRDV1*01	TRDD3*01	TRDJ1*01
CO3	CALGE LR **WP** DKLIF	TRDV1*01	TRDD3*01	TRDJ1*01
(Y)WG(I)+ Vδ1 clonotypes				
D1	CALGE TGRD **WGY** CD KLIF	TRDV1*01	TRDD3*01	TRDJ1*01
	CALGE QRS **YWG** T TDKLIF	TRDV1*01	TRDD3*01	TRDJ1*01
	CALGE LDRLFP **RWGI** I TDKLIF	TRDV1*01	TRDD3*01	TRDJ1*01
	CALGE LFL **IWGI** AY TDKLIF	TRDV1*01	TRDD3*01	TRDJ1*01
	CALGE SSSYP **YWG** TPPLY TDKLIF	TRDV1*01	TRDD3*01	TRDJ1*01
	CALGE TWG **WGI** RPPY TDKLIF	TRDV1*01	TRDD3*01	TRDJ1*01
	CALGD RNFLPS **YWGI** TY TDKLIF	TRDV1*01	TRDD3*01	TRDJ1*01
	CALGE QLTFLV **YWGM** GTPY TDKLIF	TRDV1*01	TRDD3*01	TRDJ1*01
	CALGE LSP **HWGI** LV TAQLFF	TRDV1*01	TRDD3*01	TRDJ2*01
	CALGE GVRME **YWGIR** S WDTRQMF	TRDV1*01	TRDD3*01	TRDJ3*01
D2	CALGE TSYPRS **YWG ** LY TDKLIF	TRDV1*01	TRDD3*01	TRDJ1*01
	CALGE TSYPRS **YWG** LY TDKLIF	TRDV1*01	TRDD3*01	TRDJ1*01
	CALGE LVSRRR **WGIR** GGE LIF	TRDV1*01	TRDD3*01	TRDJ1*01
	CALG IPKALHP **VWGIR** R TDKLIF	TRDV1*01	TRDD3*01	TRDJ1*01
	CALGE LAFLYV **YWGIR** PY TDKLIF	TRDV1*01	TRDD3*01	TRDJ1*01
	CALG TL **WGIR** FGYRG QLFF	TRDV1*01	TRDD3*01	TRDJ2*01

CD1-restriction mediating (Y)WG(I) motif in D3 segment are given in bold.

About half of the vaccine-reactive γ-clonotypes in both donors were TRGV1-8^+^ thus Vγ9^negative^ and their γ-chain CDR3-region homologous to CD1-reactive γδ-T cells (**Figure 5c**), and the most common TCRγ clonotype (n=12) in D2 with the exception of 1 amino acid was identical with the public CD1-restricted CO3 TCRγ-clonotype CATWDGDYYKKLF (convergent CDR3 formation). TRGV4-TRGJ1 as well as other TRGV-TRGJ rearrangements used by public CD1-reactive TCRs were present in D1 and D2 (**Table 5A**).

The significant proportion of Vγ10-clonotypes in our analysis represent most likely “unproductive” γ-alleles of peptide-responsive αβ-T cells (90), in the vaccine-reactive T-cell pool (not shown).

### Vaccine-reactive Vδ1^+^ TCR clonotypes bear the CD1-restricted WGI/Y motif

CD1-restricted γδ-TCRs contact CD1 through a Vδ1-segment containing a germline-encoded WGI/Y motif (91). WGI/Y bearing Vδ1-segments of both donors (**Figure 5b**), when aligned with public CD1-restricted Vδ1-clonotypes show strong homology with these, clearly supporting a presentation of the hydrophobic peptide LLLLDRLNQLESKMS by CD1-molecules (**Figure 5c**, **Table 6B**).

### Vδ1-clonotypes with germline-derived LGD motif assigns them to the adaptate virus-reactive γδ-T-cell compartment

D1`s Vδ1-repertoire had an additional germline-derived motif: LGD (**Figure 5b**). While the WGI/Y motif is associated with CD1 recognition, LGD containing public CDR3 sequence TRDV1-TRDD3-CALGELGD was previously shown to be expanded in CMV-infection and assumed to belong to the “adaptate” γδ-T-cell compartment, representing a radically new adaptive immunobiology (92) which displays potent cytolytic function against virally infected and malignant cells, strong cytokine production and expression of NKRs (93–95). Adaptive Vδ1Cδ-TCRs show true (pauciclonal) expansions in adult and cord blood Vδ1Cδ-TCR-repertoires, are overwhelmingly private, even more so than TCRβ (96), are apparently unrelated both within and between individuals, and their CDR3 lengths are highly variable (96).

Unsurprisingly, Vδ1Cδ-clonotypes in D1 and the non-WGI bearing clonotypes in D2 (3Vδ1Cδ/9) share these characteristics: they are private, inconsistent in CDR3 length due to a high proportion of N or (P) nucleotides (mean 19 and 22 N/P nucleotides; **Supplementary Table 2**, underlined aa), and lack a conserved motif. The most expanded Vδ1Cδ-clonotypes consistently share strong hydrophobicity in their N-nucleotide-encoded CDR3 though, with ≥50% amino acids being aliphatic combined with (1-3) basic amino acids (R,K,H) in D1 and D2.

Interestingly, motifs YWGI/Y and LGD are both encoded by TRDD3*01 and defined by the reading frame set by TRDV1*01 and D-segment breakpoints and N-nucleotide insertion (97, 98).

### Vaccine-reactive Vδ2Cδ clonotypes belong to the adaptive Vδ2-subset

Recent research redefines human Vδ2^+^-T-cell compartment by separating it into an innate (Vγ9^+^) and adaptive (Vγ9^negative^) subset with distinct functions in (microbial) immune surveillance (99). Vγ9^negative^ Vδ2^+^ cells undergo targeted clonal expansion/differentiation in response to acute viral infection in both peripheral blood and solid tissues, and thus have the ability to mount a pathogen-specific immune response like Vδ1 and αβ-T cells.

The vast majority of Vδ2-clonotypes in this study was private (**Supplementary Table 3**) with unique V(D)J rearrangements, individual CDR3-length and no relatedness to each other or to clones from the other donor, resembling Vδ1 and adaptive αβ-T cells, which undergo profound and highly focused clonal expansions from an originally diverse and private TCR-repertoire in response to specific immune challenges (100).

**Figure 6:**
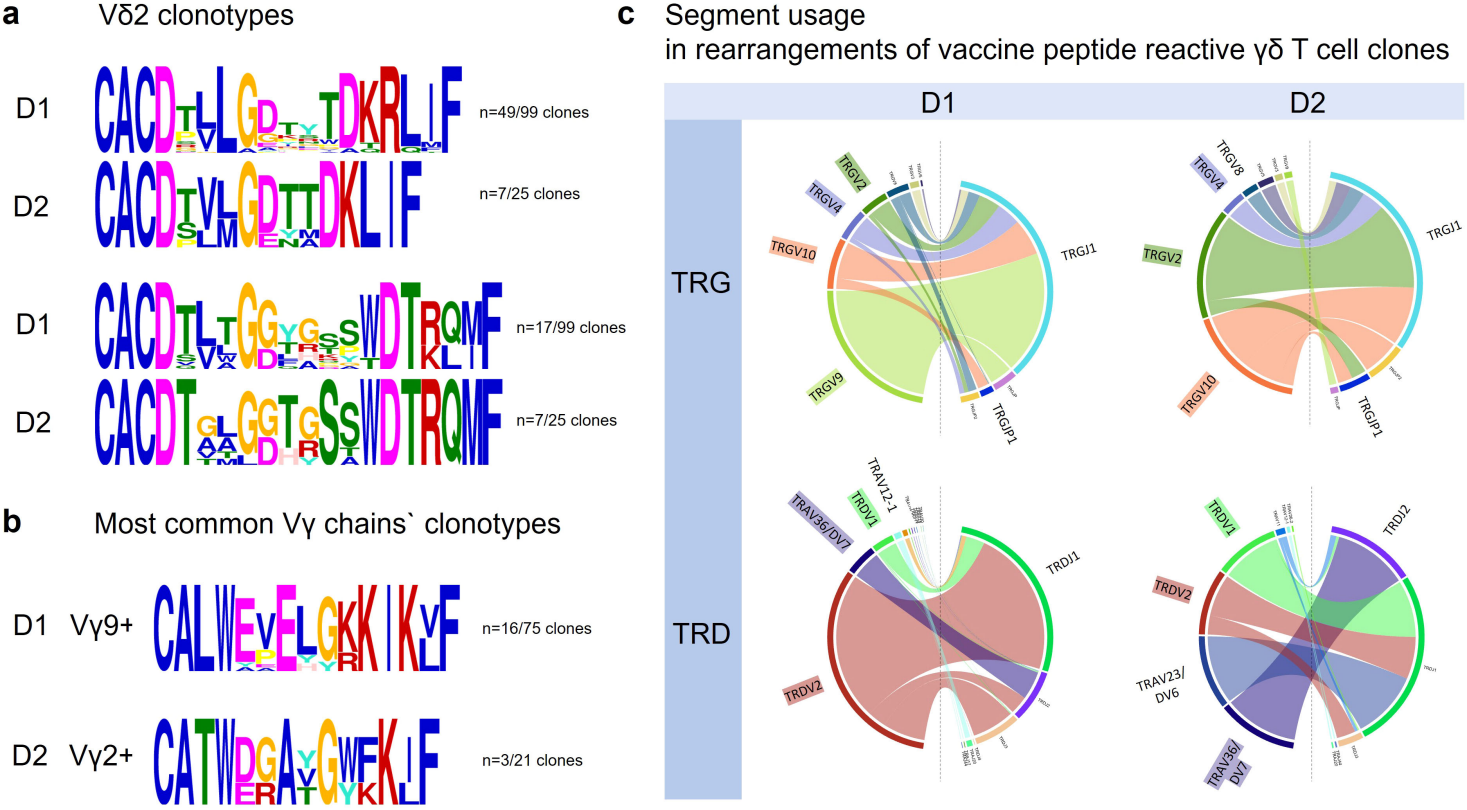
Vδ2 and Vγ clonotypes. **(a)** In Vδ2 clonotypes LGD was clearly the most frequent conserved motif (D1: n=49/99 and D2: n=7/25), ahead of GG (D1: n=17/99 D2 n=7/25). **(b)** The few but most expanded clonotypes of D1 were TRGV9+ and public and reported in context of CMV and EBV immunity (CALWEVELGKKIKVF). The most expanded peptide reactive Vγ2+ clonotypes in donor 2 are private (underlying sequences are depicted in **Table 7**). **(c)** Chord diagrams show the segments most commonly used in rearrangements in D1 and D2 vaccine reactive γ and δ clonotypes.

Vδ2^+^-clonotypes containing a LGD or GG motif were identified (**Figure 6a**)

Phosphoantigen (Pag)-reactivity of the Vδ2-clonotypes is unlikely, as corresponding Pag-reactive Vγ9-clonotypes have not been identified. Consistent with an adaptive-like vaccine-reactive Vδ2^+^-phenotype, the γ-repertoires were predominantly Vγ9^negative^ (59/75 in D1; 20/21 in D2). Of the only few Vγ9^+^ (**Figure 6b**) none was using JP1, the J-segment of Pag-reactive semi-invariant Vγ9JP1Vδ2-TCR, the most abundant γδ-TCR in adult peripheral blood (101, 102)

The few TRGV9^+^-clonotypes (two germline-encoded public sequences (CALWEVQELGKKIKVF TRGV9*01 TRGJP*01) (95, 102) published in context of CMV (103) and Epstein-Barr virus (104) and several private clonotypes (CALWYEELGKKIKVF TRGV9*01/TRGJP*01) contained the JP-segment in D1. D2 had only one single Vγ9^+^-clonotype, which was private (TRGV9*01 TRGJP*01; CALWEAIQELGKKIKVF, **Table 7**). JP1 however was identified in several Vγ9^negative^ -clonotypes (D1: n=11/D2: n=3) (**Figure 6c**). In D2, the most expanded clonotype was Vγ2^+^ TRGV2*03-CATWDGDYYKKLF, identical to the CD1a-reactive CO3 TCRγ-chain clonotype except for 1 amino acid (88).

**Table 7:** Underlying sequences of logoplots in **Figure 6b**.

TRVγ sequences			
Donor	CDR3	V	J
D1	CALWEVLGKLF	TRGV9*01	TRGJ1*02
	CALWEPHGRKKLF	TRGV9*01	TRGJ1*02
	CALWEVQELGKKIKVF	TRGV9*01	TRGJP*01
	CALWYEELGKKIKVF	TRGV9*01	TRGJP*01
	CALWEPQELGKKIKVF	TRGV9*01	TRGJP*01
	CALWEVPELGKKIKVF	TRGV9*01	TRGJP*01
	CALWEVHPSNYYKKLF	TRGV9*01	TRGJ1*02
	CALWEVQELGKKIKVF	TRGV9*01	TRGJP*01
	CALWEVQKELGKKIKVF	TRGV9*01	TRGJP*01
	CALWEVWSELGKKIKVF	TRGV9*01	TRGJP*01
	CALWAPERELGKKIKVF	TRGV9*01	TRGJP*01
	CALWEVLRKLS	TRGV9*01	TRGJ1*02
	CALWEVLGKLF	TRGV9*01	TRGJ1*02
	CALWEVLGKLF	TRGV9*01	TRGJ1*02
	CALWEPHGRKKLF	TRGV9*01	TRGJ1*02
	CALWEATYYKKLF	TRGV9*01	TRGJ1*02
D2	CATWDGYYKKLF	TRGV2*03	TRGJ1*02
	CATWDGATGWFKIF	TRGV2*03	TRGJP1*01
	CATWERVGWFKIF	TRGV2*03	TRGJP1*01

### Chord diagrams for γ and δ transcripts identify Vδ2^+^-clonotypes as members of the adaptive Vδ2^+^ T-cell subset

Chord diagrams (**Figure 6c**) do not show the number of clones, but the number of unique sequences with this rearrangement, i.e. how often a clone was represented. For example, in D1 there were only 16 TRGV9 clones, but they were very common (highly expanded) (**Figure 6c**). Important to note and as mentioned above: none of these 16 TRGV9 clones used the segment JP1, that segment which is part of the phosphoreactive Vδ2Vγ9 TCRs (**Figure 6C**). Only one Vγ9 clonotypes was detected in D2.

Taken together, TCR bulk sequencing does not allow for the identification of heterodimers, yet the γ-chain clonotypes detected were mainly Vγ9^negative^ (59/75 and 20/21 γ in D1 and D2 respectively) and almost exclusively private. The few Vγ9^+^ clonotypes did *not* show JP1 prerequisite for Pag-sensing. Therefore, from a purely statistical point of view, this supports the assumption that Vδ2^+^-clonotypes identified in this study belong to the adaptive Vγ9^negative^Vδ2^+^-T-cell subset (99).

### Atypical T-cell clonotypes (γδ, VαCδ, VδCα)

The γδ-T-cell clone HCSHFLDPYSALCV and Vδ/C**α** clone VLPALLSQTQLGSFPPLRP belong to the overlap repertoire and have atypical CDR3 regions (**Tables 4**, **8**). They lack the conserved Candeias cysteine and the CALGE/CLV motif, but have exceptionally long stretches of N-nucleotides between V and D and D and J segments (D1: 58 and 22, D2: 22 and 8 respectively). Interestingly, the VLPALLSQTQLGSFPPLRP (TRAV23/DV6*01, TRDD2*01, TRDJ4*01, Cα; **Tables 4**, **8**) clonotype is annotated in context of T-cell reconstitution in pediatric patients after HSCT using TCRαβ/CD19-depleted hematopoietic cell grafts, and derived from unproductive TRBV-rearrangements (patient 1: TRBV1*01 TRBD1*01 TRBJ2-3*01 (3 different clone sequences) (Patient 2: TRBV1*01 TRBD2*01 TRBJ2-3*01). HCSHFLDPYSALCV (TRAV36/DV7*01, TRDD2*01, TRDJ2*01, Cδ) was not annotated in public databases but was the most frequent γδ-T-cell clone of both donors with 140 and 50 unique sequences in D1 and D2 respectively. Being in the overlapping repertoire makes the clone “public”.

**Table 8:** Exotic and hybrid γδTCRs including VδCα, VαJCδ, and atypical VδCδ clonotypes.

Exotic rearrangement	CDR3 sequence	Hydrophobic amino acids in CDR3	Public
Hybrid γδTCR (Vδ6/Cα)	**VLPALLSQTQLGSFPPLRP**	12/19	present in both donors, superpublic
Hybrid γδTCR (Vα12/J44/Cδ)	**LPVSF**	4/5	present in both donors, superpublic
Hybrid γδTCR (Vα12/J20/Cδ)	**LPVSF**	4/5	present in both donors, superpublic
γδTCR (Vδ7/Cδ)	**HCSHFLDPYSALC**	8/14	present in both donors, public

Aliphatic amino acids are marked in green, basic amino acids in red.

CDR3 regions are given in bold.

### Vα-Cδ TCRs

The two Vα12/Cδ clones, both identified in both donors (**Tables 4**, **8**) resulted from differing underlying rearrangements involving Jα22 or Jα44 fused to TRAV12 respectively. Their CDR3 sequence LPVSF is commonly found in public databases yet annotated for TRAV CDR3 in CD4^+^ T cells αβTCRs (79, 80) and has been identified in-house in Glioblastoma TIL and γδ cells from healthy donor peripheral blood, albeit each time as VαCδ hybrid clonotypes though (unpublished data).

The Cδ-segment in the Vα-Cδ hybrid clonotype can only pair with a γ-chain to form a γδTCR that recognizes its targets directly or presented by different anchor molecules (81).

## Discussion

This study reveals a multi-layered immune response elicited by a single 15-mer aliphatic peptide in two individuals. The small sample size of two individuals are case-level observations and thereby restrict the strength of the conclusions, however this approach reflects the exploratory and descriptive nature of this study while highlighting important clues and patterns worthy of further investigation. A follow-up study will employ more targeted approaches like single-cell TCR sequencing and functional assays of peptide-specific T-cells as well as functional capacity of γδ- and hybrid clonotypes and will be the next important step to build on our molecular findings in this study.

Designed specifically for promiscuous MHC class II binding, LLLLDRLNQLESKMS recognizes the important role CD4^+^ T cells play in immune responses to neoantigens (105) and enabled us to study its immunogenicity in the context of allogeneic T cells. We found a similarly robust immune response in both donors following the same principles. The aliphatic nature of the peptide, allowing presentation also via CD1, added a layer of pre-programmed immunity to the adaptive responses. Although using only a small fraction of the cDNA used to sequence adaptive TCRs, we found an abundance of unconventional T cells including (CD1-restricted) Vδ1-, adaptive Vδ2-, and VαCδ clonotypes, complementing the wealth of adaptive T-cell clonotypes.

This finding is significant since γδ-t cells make up the majority of T cells in epithelia where viruses enter the body and are key in the induction and orchestration of the early immune response.

Overlap between the repertoires was small: 15 TRA and 9 TRB clonotypes, one γδ T-cell clone HCSHFLDPYSALCV, and in addition two VαCδ (with differing underlying rearrangement) and one VδCα clonotype were found in both donors.

Although representing only a small percentage of the total sequences, the overlap repertoire reveals interesting features: the adaptive clonotypes are not homologous to each, i.e. show neither convergent CDR3 formation nor conserved motifs, and have different CDR3 lengths. The γδ-T-cell clone is atypical, lacking a characteristic 5` CALGE/CACD but showing a large number of N nucleotides, which is more characteristic of conventional adaptive TCR clonotypes. In contrast, clonotypes of adaptive immunity shared between the donors show few or no N nucleotides, identifying them as TdT-independent “neonatal” TCRs.

CDR3-length-fragment analysis with an almost Gaussian profile in the α, β and δ repertoires did not suggest pronounced clonal expansions, nor did clonotype analysis of the most commonly represented CDR3 length fragments. However, the clonotypes examined shared N-nucleotide encoded aliphatic amino acids in their mostly unique CDR3 binding region. Tree plots confirmed the finding of predominantly unique peptide-responsive CDR3 clonotypes in the α and β sequences.

Based on the finding that physicochemical features of the germline-encoded CDR1 and CDR2 regions of the Vα and β segments contribute to the specificity of a TCR (62), we identified immunodominant rearrangements in the immune repertoires (**Figure 3c**). Chord diagrams show the restricted use of a few, i.e. 6 out of a total of 64 TRAV/TRBV-segments used in approximately half of all α and 4, respectively 8/47 functional TRBV-segments in the β-clonotypes in both donors, with several of the segments being identical in both donors. Not surprisingly, the CDR2 regions of the immunodominant segments show an identical germline-encoded motif (SN) (examined for TCRα chains only), their germline-encoded CDR1 regions concordantly harboring up to 5 aliphatic amino acids, strongly suggesting - in analogy to recent work - that these will contact the MHC-presented aliphatic peptide. Finally, the CDR3 regions corresponding to the preferred V-segments consistently show abundant N-nucleotide encoded *aliphatic* amino acids but lack a conserved motif.

These findings are highly consistent with studies by Greenshields-Watson et al, Gao et al, and Wang et al, who concordantly report a TCR-V segment bias in virus-responsive repertoires in infants, adults, and individuals with elite control of HIV, that is based on germline-encoded TCR-MHC contacts with complementary biochemical features of TCR and MHC molecules (62, 100, 106), which is indeed intriguing.

Vδ1Cα clonotypes from this study were compared with previously published adaptive immunity clonotypes, and matched exclusively with germline-encoded portions of annotated Covid-19 reactive adaptive T cells, “motifs” that were also shared between our donors.

The reverse approach, i.e. stripping the germline sequences from the V-(D)-J CDR3 regions of the clonotypes, i.e. reducing them to purely N-nucleotide encoded segments, did not lead to the identification of motifs, but highlighted the conservation of their charges for hydrophobicity, both in the α, β and Vδ1Cα and even the Vδ1Cδ clonotypes.

N-encoded hydrophobic residues as a common feature of all CDR3 binding regions reactive to the vaccine-peptide focus attention on the peptide. Chowell et al. show that hydrophobicity of TCR contact residues is a hallmark of immunogenic epitopes (107). The correlation between hydrophobicity and immunogenicity is so clear that empirical testing is unnecessary (107). The broad clonotype repertoires induced in both donors by the aliphatic vaccine-peptide strongly support that hydrophobicity is decisive for the immunogenicity of a peptide.

Kanduc et al. indirectly confirm Chowell`s statement by showing that hydrophilicity and hydrophobicity characterize the tolerated common peptide sequences and the immunogenic rare peptides, thus forming the physicochemical basis of immunotolerance (108).

Their outstanding findings provide a straightforward rationale for vaccine-peptide development.

Indeed, hydrophobic peptides as a promising approach for vaccine development are already suggested by the fact that the nucleocapsid-derived LLLLDRLNQLESKMS is potentially associated with pre-existing T-cell specificities (previous coronavirus responses). The combination of immunogenic hydrophobicity with promiscuous MHC class II-binding is even more promising, as it elicits responses that are noteworthy in several ways: induction of pre-programmed Vδ1 γδ-clonotypes, Vδ2-clonotypes that can be considered as recruited mainly from the adaptive, Vδ2Vγ9^negative^ compartment according to the gamma-repertoire, adaptive Vδ1Cα-hybrid-TCRs endowed with immediate, innate properties, and “neonatal”, almost germline-encoded TCRs, which together may constitute a kind of “early sensing” that facilitates adaptive immune responses by preparing the milieu for their initiation and by broadening the scope with adaptive-like responses. At the same time, the α- and β-clonotypes detected in the study - maximally diverse but all reactive to this one peptide - do not correspond to the prototype of an adaptive immune response, at least not the one we know from class I restricted peptides: namely, the induction and strong expansion of several different clones characterized by convergent CDR3 formation and/or motif conservation.

In contrast, the αβ-T cells in this study tend to exhibit features characteristic of innate T cells: few/no N-nucleotides, preferential V-segment usage with their germline-encoded CDR1 and CDR2 showing preferences for aliphatic/hydrophobic targets - and thus appear to be pre-programmed in their ‘specificity’ - no focus on a few clones, but rather a large collection of unique clonotypes that share one characteristic, their hydrophobic N-nucleotide encoded amino acids in their CDR3 epitope binding region.

Functionality and specificity of VδCα-hybrid clonotypes was described recently by Pellicci et al. (109) who show that TCRs comprised of a TCR-δ variable gene (Vδ1) fused to joining α and constant α domains, paired with an array of TCR-β chains represent ∼50% of all Vδ1^+^ human T cells, which can recognize peptide- and lipid-based Ags presented by human leukocyte antigen (HLA) and CD1d. Thus VδCα-hybrid clonotypes confer Ag specificity beyond classical understanding of T cell biology and TCR diversity.

Seminal studies by Legut et al. (110) provide molecular and cellular evidence for the productivity of VδCα-hybrid clonotypes. Both groups demonstrate robust cell surface expression of Vδ1/Jα/Cα TCRchains. Notably, they found these rearrangements in polyclonal, IL-2–driven human T cell lines and healthy donor repertoires, with broad Jα usage—a finding that strongly supports their functional expression.

Also, Volkmar et al. (111) identified human T cell receptors harboring TRDV genes within the TRA chain. This provides additional confirmation of productive rearrangements and surface expression of Vδ-Jα-Cα molecules in human and murine T cell repertoires. These findings corroborate those of Miossec et al. (112), substantiating the reproducibility and biological relevance of these hybrid TCRs across different experimental platforms and donor sources.

That hybrid Vδ-Jα-Cα chains can form functional TCR heterodimers with TCRß chains was evidenced by Volkmar et al. (111) using stainings with the monoclonal antibody BMA031 specifically recognizing a determinant in the constant region of the TCR beta chain. This specificity for the TCR beta constant region underlies its use in discriminating αβ T cells from γδ T cells.

Pellici et al. (109), Legut et al. (110), Miossec et al. (112) and Volkmar et al. (111) all propose that VδCα-hybrid clonotypes may enable unique antigen recognition distinct from typical αβ or γδ TCRs, and that these hybrid TCRs remain areas of active investigation.

Critical consideration should be given to the involvement of γδ-T cells in the immune response. The germline-encoded CDR1 and 2 regions of the Vα in **Vα12/Cδ** hybrid-clonotypes suggest MHC class II-restriction/association, and since γδ-TCRs can also recognize MHC class II directly, even independently of antigen, one can indeed speculate that these TCRs recognize their antigen in an MHC class II-restricted manner.

Also another MHC class II-associated recognition-scenario is conceivable: the MHC-binding groove accommodates peptides based on the formation of conserved hydrogen bonds between the side chains of the MHC molecule and the backbone of the peptide and the occupation of defined pockets by peptide side chains, with anchor residues P1, P4, P6 and P9 in MHC class II (113–116).

While the MHC class I-binding groove is closed, MHC class II is open at both ends, thereby allowing longer peptides or even intact proteins to bind (13–25 peptides, average length is 15 (117)) and to be loaded onto MHC class II (118, 119).

This allows peptide protrusions from the groove at the NH2 and COOH termini, commonly known as peptide flanking regions (PFRs), which vary in length and composition but have a significant impact on immunodominance/immunogenicity and subsequent T-cell interaction (120–123).

Most interestingly, most immunogenic epitopes generate CD4^+^ T cells that are dependent on these MHC class II-linked peptide flanking residues, and naturally processed HLA class II peptides show highly conserved immunogenic flanking region sequence preferences with a key role for lipophilic residues. In light of this, it seems reasonable to speculate that PFRs may also be targeted by innate immune cells, and recognition of a hydrophobic protruding residue of the vaccine-peptide LLLLDRLNQLESKMS may lie within the multifunctional scope of γδ-T cells. This would be consistent with the fact that γδ-TCRs can recognize MHC class II regardless of the peptide presented and regardless of the specificity of the molecule presented (85). And, it would be compatible with the binding properties of VαCδ hybrid-TCRs.

The recognition of hydrophobic residues of peptides by true γδ-T cells has long been known. Mycobacteria HSP-60-specific γδ-TCRs structural requirements for stimulation related to the smallest stimulatory mycobacterial HSP-60 peptide **F**G**L**QLEL (HSP-60 positions 181-187), are the 5` hydrophobic residues phenylalanine (F) and leucine (L) (positions 181 and 183), in a non-conserved region among HSP-60 molecules of other species, thus γδ-T cells mediate an epitope and pathogen specific immune response (124).

Remarkably, the unconventional Vδ6Cα, the Vδ7/Cδ, and the hybrid VαCδ clonotypes of the overlap repertoire (**Tables 4**, **8**), all exhibit significant hydrophobicity with predominantly aliphatic and non-polar aa in their CDR3 regions (**Table 8**) pointing to a binding preference for hydrophobic moieties.

### γδ-T cells can recognize (peptide-loaded) MHC classes I and II

γδ-TCR-ligand recognition is a means by which γδ-T cells discriminate between homeostasis and stress conditions (125).

That this includes MHC class I and II molecules recognition was shown for human HLA- A2 (126), HLA-A24 (127), HLA-B27 (128), which can specifically activate/expand human γδ-T-cell clones from healthy individuals in culture, independent of peptide presentation, thereby γδ-T-cells recognize conserved parts of the MHC. These findings were expanded by showing that the Vγ5Vδ1^+^TCR – which recognizes HLA-A*24:02 on cancer cells with a key role for CDR1 and CDR2 in MHC binding - *is* dependent on peptide loading of the HLA complex but *not* the presentation of a *specific* peptide (125). Peptide-presenting MHC-molecules have increased stability on cell surfaces, thus random peptide presentation may confer target stabilization rather than specific antigen presentation.

Finally, direct interaction of expanded Vδ1^+^ TCRs with the MHC-II-complex and massive expansion of individual Vδ1^+^ γδ-T cell clones during viral infection were described (85).

In this context another report is significant (83). Vδ1^+^-γδ-T cells derived *in vitro* from human hematopoietic stem and progenitor cell (HSPC) can react with and expand in response to HLA-A2-presented melanoma antigen MART-1. The binding of the respective γδ-TCRs to MART-1-pMHC is less peptide-centric as compared to the interaction with a MART-1-specific αβ-TCR and it is speculated that MART-1 may act as specific stabilizer for the MHC for proper recognition by the respective γδ-TCRs. Intriguingly, the heteroclite peptide MART-1-(26-35) ELAGIGILTV is highly aliphatic: 8/10 amino acids are hydrophobic (83).

### The potential of induced unconventional T-cell responses

A vaccines potential of inducing unconventional T-cell responses offers substantial immunologic and therapeutic potential by bridging innate and adaptive immunity, particularly at barrier sites where pathogens first contact the host. Unconventional T cells, such as γδ T cells, recognize a broad spectrum of antigens, including non-peptide metabolites and lipids presented by non-classical MHC molecules. This enables them to rapidly respond in an innate-like manner, offering early defense that can contain infections before conventional T cells are fully activated (129).

γδ T cells are preferentially localized at epithelial barrier surfaces—such as the respiratory, gastrointestinal, and urogenital tracts—the main viral entry points. Here, they contribute to barrier immunity by detecting conserved microbial and stress-induced ligands, and as we know according to Davey et al. (96, 99) and the present study: also CD1 or MHC class II presented ligands, promoting local immune responses, maintaining tissue homeostasis, and facilitating repair after injury (96, 99, 130–132). Their capacity for rapid cytokine secretion and cytotoxicity complements physical epithelial defenses and innate immune sensing, enhancing early viral control (133).

Clinically, harnessing unconventional T-cell responses opens new avenues for vaccines and immunotherapies that provide rapid, broad-spectrum protection, especially against pathogens like viruses that exploit barrier tissues. Their relative resistance to exhaustion and pre-expanded tissue presence makes them attractive for therapies targeting infections, but also cancer, and inflammatory diseases (134). Overall, unconventional T cells augment the immune system’s plasticity and robustness, particularly at the critical interfaces between host and environment.

In conclusion, the immune response elicited by a single highly aliphatic vaccine peptide, predicted to promiscuously bind to MHC class II and according to its hydrophobic properties is presentable via CD1, seems to suggest two things: First, that the boundaries between adaptive and innate immunity may be more fluid than previously thought, and that αβ-T cells, which represent the end of an evolutionary process toward specificity, may rely on/cooperate with the intermediate stages of this process – the contribution of innate and hybrid-TCR-bearing T cells - which may contribute non-redundantly but independently to the induction, establishment, and consolidation of specificity, the cornerstone for the formation of long-term memory.

## Data Availability

The subjects’ repertoire data are publicly available as part of the AIRR Data Commons on VDJ Server (https://vdjserver.org/community?study_id=PRJNA1232000). The raw fastq sequencing files have been deposited in NCBI’s Sequence Read Archive (SRA) and are accessible under the BioProject PRJNA1232000 through the SRA accession numbers SRR32574224 and SRR32574225.
