# Correction: Promiscuous class II-binding SARS-CoV-2-nuc derived vaccine-peptide induced extensive conventional, innate and unconventional T cell responses

**DOI:** 10.3389/fimmu.2026.1788957

**Published:** 2026-02-13

**Authors:** Naomi Krickeberg, Hans-Georg Rammensee, Karin Schilbach

**Affiliations:** 1 Department of Pediatric Hematology and Oncology, University Children’s Hospital Tübingen, Tübingen, Germany; 2 Institute of Immunology, University of Tübingen, Tübingen, Germany; 3 German Cancer Consortium (DKTK), partner site Tübingen, a partnership between DKFZ and University Hospital Tübingen, Germany, Cluster of Excellence iFIT (EXC2180) “Image-Guided and Functionally Instructed Tumor Therapies”, University of Tübingen, Tübingen, Germany

**Keywords:** SARS-CoV-2, peptide vaccine, SARS-CoV-2 nucleoprotein peptide, T cell response, unconventional T cells, adaptive Vδ2γ9^negative^ γδ T-cells, CD1 restricted, vaccine peptide reactive Vδ1 γδ T-cells

The vaccine peptide was incorrectly described as a 16-mer; it should be referred to as a 15-mer. A correction has been made to the section Discussion: “This study reveals a multi-layered immune response elicited by a single 15-mer aliphatic peptide in two individuals”

The peptide sequence and length were incorrectly stated as the 16-mer "LLLLDRLLNQLESKMS" throughout the manuscript. This has been corrected to the accurate 15-mer vaccine peptide sequence, "LLLLDRLNQLESKMS", in all relevant instances.

The reference for “For the same TCRs, it is shown that the germline- encoded parts of the CDR3 region of the TCRα chain do not contact the target peptide, but only one or a few N-nucleotide encoded amino acids which, very importantly, are identical or charge-conserved in TCRs of the same specificity. Successive studies showed the same result” was erroneously written as:

46. Gabernet G, Marquez S, Bjornson R, Peltzer A, Meng H, Aron E, et al. nf-core/ airrflow: An adaptive immune receptor repertoire analysis workflow employing the Immcantation framework. *PloS Comput Biol*. (2024) 20:e1012265. doi: 10.1371/journal.pcbi.1012265

47. Di Tommaso P, Chatzou M, Floden EW, Barja PP, Palumbo E, Notredame C. Nextflow enables reproducible computational workflows. *Nat Biotechnol*. (2017) 35:316–9. doi: 10.1038/nbt.3820

It should be:

63. Ritmahan W, Kesmir C, Vroomans RMA. Revealing factors determining immunodominant responses against dominant epitopes. *Immunogenetics*. (2020) 72:109-18.

64. Stern MH, Lipkowitz S, Aurias A, Griscelli C, Thomas G, Kirsch IR. Inversion of chromosome 7 in ataxia telangiectasia is generated by a rearrangement between T-cell receptor beta and T-cell receptor gamma genes. *Blood*. (1989) 74:2076-80.

The reference for “Recent studies have shown that the adaptive TCR repertoire consists of two ontogenetically and functionally distinct TCR types, which are regulated by variations in thymic production and terminal deoxynucleotidyl transferase (TDT) activity.” was erroneously written as:

27. McBride R, van Zyl M, Fielding BC. The coronavirus nucleocapsid is a multifunctional protein. *Viruses*. (2014) 6:2991–3018. doi: 10.3390/v6082991

It should be:

66. Trofimov A, Brouillard P, Larouche JD, Seguin J, Laverdure JP, Brasey A, et al. Two types of human TCR differentially regulate reactivity to self and non-self antigens. *iScience*. (2022) 25:104968.

The reference for “Strikingly, the CDR3 sequences of the overlap repertoire and the most common vaccine-reactive αβ T clonotypes (**Table 5**) lack or have only few N nucleotides, assigning them to the neonatal TCRs derived from TDT-negative precursors, which persist throughout life, are highly shared between individuals and are reported to be disease-associated.” was erroneously written as:

27. McBride R, van Zyl M, Fielding BC. The coronavirus nucleocapsid is a multifunctional protein. *Viruses*. (2014) 6:2991–3018. doi: 10.3390/v6082991

It should be:

66. Trofimov A, Brouillard P, Larouche JD, Seguin J, Laverdure JP, Brasey A, et al. Two types of human TCR differentially regulate reactivity to self and non-self antigens. *iScience*. (2022) 25:104968.

The reference for “The vast majority of Vδ2-clonotypes in this study was private (**Supplementary Table 3**) with unique V(D)J rearrangements, individual CDR3-length and no relatedness to each other or to clones from the other donor, resembling Vδ1 and adaptive αβ-T cells, which undergo profound and highly focused clonal expansions from an originally diverse and private TCR-repertoire in response to specific immune challenges” was erroneously written as:

84. Benveniste PM, Nakatsugawa M, Nguyen L, Ohashi PS, Hirano N, Zuniga- Pflucker JC. *In vitro*-generated MART-1-specific CD8 T cells display a broader T-cell receptor repertoire than ex vivo naive and tumor-infiltrating lymphocytes. *Immunol Cell Biol*. (2019) 97:427–34. doi: 10.1111/imcb.2019.97.issue-4

It should be:

100. Wang Y, Tsitsiklis A, Devoe S, Gao W, Chu HH, Zhang Y, et al. Peptide Centric Vbeta Specific Germline Contacts Shape a Specialist T Cell Response. *Front Immunol*. (2022) 13:847092.

The reference for “That this includes MHC class I and II molecules recognition was shown for human HLA- A2 (33), HLA-24 (31), HLA-B27 (32)]” was erroneously written as:

33. Hu Y, Petroni GR, Olson WC, Czarkowski A, Smolkin ME, Grosh WW, et al. Immunologic hierarchy, class II MHC promiscuity, and epitope spreading of a melanoma helper peptide vaccine. *Cancer Immunol Immunother*. (2014) 63:779–86.

31. Heitmann JS, Tandler C, Marconato M, Nelde A, Habibzada T, Rittig SM, et al. Phase I/II trial of a peptide-based COVID-19 T-cell activator in patients with B-cell deficiency. *Nat Commun*. (2023) 14:5032. doi: 10.1038/s41467-023-40758-0

32. Ochoa R, Lunardelli VAS, Rosa DS, Laio A, Cossio P. Multiple-Allele MHC Class II Epitope Engineering by a Molecular Dynamics-Based Evolution Protocol. *Front Immunol*. (2022) 13:862851. doi: 10.3389/fimmu.2022.862851

It should be:

126. Spits H, Paliard X, Engelhard VH, de Vries JE. Cytotoxic activity and lymphokine production of T cell receptor (TCR)-alpha beta+ and TCR-gamma delta+ cytotoxic T lymphocyte (CTL) clones recognizing HLA-A2 and HLA-A2 mutants. Recognition of TCR-gamma delta+ CTL clones is affected by mutations at positions 152 and 156. *J Immunol*. (1990) 144:4156-62

127. Ciccone E, Viale O, Pende D, Malnati M, Battista Ferrara G, Barocci S, et al. Specificity of human T lymphocytes expressing a gamma/delta T cell antigen receptor. Recognition of a polymorphic determinant of HLA class I molecules by a gamma/delta clone. *Eur J Immunol*. (1989) 19:1267-71.

128. Del Porto P, D’Amato M, Fiorillo MT, Tuosto L, Piccolella E, Sorrentino R. Identification of a novel HLA-B27 subtype by restriction analysis of a cytotoxic gamma delta T cell clone. *J Immunol*. (1994) 153:3093-100.

The reference for “Here, they contribute to barrier immunity by detecting conserved microbial and stress-induced ligands, and as we know according to Davey et al. (95, 98)” was erroneously written as:

95. Dimova T, Brouwer M, Gosselin F, Tassignon J, Leo O, Donner C, et al. Effector Vgamma9Vdelta2 T cells dominate the human fetal gammadelta T-cell repertoire. *Proc Natl Acad Sci U S A*. (2015) 112:E556–65.

98. Sanchez Sanchez G, Papadopoulou M, Azouz A, Tafesse Y, Mishra A, Chan JKY, et al. Identification of distinct functional thymic programming of fetal and pediatric human gammadelta thymocytes via single-cell analysis. *Nat Commun*. (2022) 13:5842.

It should be:

96. Davey MS, Willcox CR, Joyce SP, Ladell K, Kasatskaya SA, McLaren JE, et al. Clonal selection in the human Vdelta1 T cell repertoire indicates gammadelta TCR-dependent adaptive immune surveillance. *Nat Commun*. (2017) 8:14760.

99. Davey MS, Willcox CR, Hunter S, Kasatskaya SA, Remmerswaal EBM, Salim M, et al. The human Vdelta2(+) T-cell compartment comprises distinct innate-like Vgamma9(+) and adaptive Vgamma9(-) subsets. *Nat Commun*. (2018) 9:1760.

As a result of the above changes, the following references have been reordered:

The reference for “The CDR2-loop of the Vδ1-segments is described to interact with lipid-presenting CD1 molecules” was erroneously written as:

63. Uldrich AP, Le Nours J, Pellicci DG, Gherardin NA, McPherson KG, Lim RT, et al. CD1d-lipid antigen recognition by the gammadelta TCR. *Nat Immunol*. (2013) 14:1137–45. doi: 10.1038/ni.2713

It should be:

65. Uldrich AP, Le Nours J, Pellicci DG, Gherardin NA, McPherson KG, Lim RT, et al. CD1d-lipid antigen recognition by the gammadelta TCR. *Nat Immunol*. (2013) 14:1137-45.

The reference for “In contrast to the TDT-negative precursors, TDT-dependent TCRs with distinct structural features and less shared among subjects” was erroneously written as:

64. Trofimov A, Brouillard P, Larouche JD, Seguin J, Laverdure JP, Brasey A, et al. Two types of human TCR differentially regulate reactivity to self and non-self antigens. *iScience*. (2022) 25:104968. doi: 10.1016/j.isci.2022.104968

It should be:

66. Trofimov A, Brouillard P, Larouche JD, Seguin J, Laverdure JP, Brasey A, et al. Two types of human TCR differentially regulate reactivity to self and non-self antigens. *iScience*. (2022) 25:104968.

The reference for “To analyze the entirety of αβ-clonotypes in a biologically meaningful way” was erroneously written as:

65. Kockelbergh H, Evans S, Deng T, Clyne E, Kyriakidou A, Economou A, et al. Utility of Bulk T-Cell Receptor Repertoire Sequencing Analysis in Understanding Immune Responses to COVID-19. *Diagnostics (Basel)*. (2022) 12. doi: 10.3390/diagnostics12051222

66. Foers AD, Shoukat MS, Welsh OE, Donovan K, Petry R, Evans SC, et al. Classification of intestinal T-cell receptor repertoires using machine learning methods can identify patients with coeliac disease regardless of dietary gluten status. *J Pathol*. (2021) 253:279–91. doi: 10.1002/path.v253.3

67. Glanville J, Huang H, Nau A, Hatton O, Wagar LE, Rubelt F, et al. Identifying specificity groups in the T cell receptor repertoire. *Nature*. (2017) 547:94–8. doi: 10.1038/nature22976

It should be:

67. Kockelbergh H, Evans S, Deng T, Clyne E, Kyriakidou A, Economou A, et al. Utility of Bulk T-Cell Receptor Repertoire Sequencing Analysis in Understanding Immune Responses to COVID-19. *Diagnostics (Basel)*. (2022) 12.

68. Foers AD, Shoukat MS, Welsh OE, Donovan K, Petry R, Evans SC, et al. Classification of intestinal T-cell receptor repertoires using machine learning methods can identify patients with coeliac disease regardless of dietary gluten status. *J Pathol*. (2021) 253:279-91.

69. Glanville J, Huang H, Nau A, Hatton O, Wagar LE, Rubelt F, et al. Identifying specificity groups in the T cell receptor repertoire. *Nature*. (2017) 547:94-8.

The reference for “To identify consensus criteria of vaccine-reactive clonotypes we followed Ritmahan et al. who showed that factors that determine whether a response becomes immunodominant (ID) per donor is that their CDR3 regions distinctively show hydrophobic aa residues compared to the subdominant (SD) responses.” was erroneously written as:

68. Ritmahan W, Kesmir C, Vroomans RMA. Revealing factors determining immunodominant responses against dominant epitopes. *Immunogenetics* (2020) 72:109–18.

It should be:

63. Ritmahan W, Kesmir C, Vroomans RMA. Revealing factors determining immunodominant responses against dominant epitopes. *Immunogenetics*. (2020) 72:109-18.

The reference for “Classically TCR-chains are encoded by genes formed by elements belonging to the same locus. However, transrearrangements between V(D)JC elements belonging to different TCR-chain loci have been described” was erroneously written as:

69. Stern MH, Lipkowitz S, Aurias A, Griscelli C, Thomas G, Kirsch IR. Inversion of chromosome 7 in ataxia telangiectasia is generated by a rearrangement between T-cell receptor beta and T-cell receptor gamma genes. *Blood*. (1989) 74:2076–80. doi: 10.1182/blood.V74.6.2076.2076

70. Lipkowitz S, Stern MH, Kirsch IR. Hybrid T cell receptor genes formed by interlocus recombination in normal and ataxia-telangiectasis lymphocytes. *J Exp Med*. (1990) 172:409–18. doi: 10.1084/jem.172.2.409

71. Kobayashi Y, Tycko B, Soreng AL, Sklar J. Transrearrangements between antigen receptor genes in normal human lymphoid tissues and in ataxia telangiectasia. *J Immunol*. (1991) 147:3201–9. doi: 10.4049/jimmunol.147.9.3201

72. Tycko B, Coyle H, Sklar J. Chimeric gamma-delta signal joints. Implications for the mechanism and regulation of T cell receptor gene rearrangement. *J Immunol*. (1991) 147:705–13. doi: 10.4049/jimmunol.147.2.705

73. Tycko B, Palmer JD, Sklar J. T cell receptor gene trans-rearrangements: chimeric gamma-delta genes in normal lymphoid tissues. *Science*. (1989) 245:1242–6. doi: 10.1126/science.2551037

It should be:

64. Stern MH, Lipkowitz S, Aurias A, Griscelli C, Thomas G, Kirsch IR. Inversion of chromosome 7 in ataxia telangiectasia is generated by a rearrangement between T-cell receptor beta and T-cell receptor gamma genes. *Blood*. (1989) 74:2076-80.

70. Lipkowitz S, Stern MH, Kirsch IR. Hybrid T cell receptor genes formed by interlocus recombination in normal and ataxia-telangiectasis lymphocytes. *J Exp Med*. (1990) 172:409-18.

71. Kobayashi Y, Tycko B, Soreng AL, Sklar J. Transrearrangements between antigen receptor genes in normal human lymphoid tissues and in ataxia telangiectasia. *J Immunol*. (1991) 147:3201-9.

72. Tycko B, Coyle H, Sklar J. Chimeric gamma-delta signal joints. Implications for the mechanism and regulation of T cell receptor gene rearrangement. *J Immunol*. (1991) 147:705-13.

73. Tycko B, Palmer JD, Sklar J. T cell receptor gene trans-rearrangements: chimeric gamma-delta genes in normal lymphoid tissues. *Science*. (1989) 245:1242-6.

The reference for “Of the only few Vg9+ (**Figure 6b**) none was using JP1, the J-segment of Pag-reactive semi-invariant Vg9JP1Vd2-TCR, the most abundant γδ-TCR in adult peripheral Blood” was erroneously written as:

100. Willcox CR, Davey MS, Willcox BE. Development and Selection of the Human Vgamma9Vdelta2(+) T-Cell Repertoire. *Front Immunol*. (2018) 9:1501. doi: 10.3389/fimmu.2018.01501

101. Delfau MH, Hance AJ, Lecossier D, Vilmer E, Grandchamp B. Restricted diversity of V gamma 9-JP rearrangements in unstimulated human gamma/delta T lymphocytes. *Eur J Immunol*. (1992) 22:2437–43. doi: 10.1002/eji.1830220937

It should be:

101. Willcox CR, Davey MS, Willcox BE. Development and Selection of the Human Vgamma9Vdelta2(+) T-Cell Repertoire. *Front Immunol*. (2018) 9:1501.

102. Delfau MH, Hance AJ, Lecossier D, Vilmer E, Grandchamp B. Restricted diversity of V gamma 9-JP rearrangements in unstimulated human gamma/delta T lymphocytes. *Eur J Immunol*. (1992) 22:2437–43. doi: 10.1002/eji.1830220937

The reference for “The few TRGV9+-clonotypes (two germline-encoded public sequences CALWEVQELGKKIKVF TRGV9*01 TRGJP*01) (95, 101) published in context of CMV (102) and Epstein-Barr virus (103) and several private clonotypes (CALWYEELGKKIKVF TRGV9*01/TRGJP*01) contained the JP-segment in D1.” was erroneously written as:

95. Dimova T, Brouwer M, Gosselin F, Tassignon J, Leo O, Donner C, et al. Effector Vgamma9Vdelta2 T cells dominate the human fetal gammadelta T-cell repertoire. *Proc Natl Acad Sci U S A*. (2015) 112:E556–65.

101. Delfau MH, Hance AJ, Lecossier D, Vilmer E, Grandchamp B. Restricted diversity of V gamma 9-JP rearrangements in unstimulated human gamma/delta T lymphocytes. *Eur J Immunol*. (1992) 22:2437–43. doi: 10.1002/eji.1830220937

102. Arruda LCM, Gaballa A, Uhlin M. Graft gammadelta TCR Sequencing Identifies Public Clonotypes Associated with Hematopoietic Stem Cell Transplantation Efficacy in Acute Myeloid Leukemia Patients and Unravels Cytomegalovirus Impact on Repertoire Distribution. *J Immunol*. (2019) 202:1859–70. doi: 10.4049/jimmunol.1801448

103. Djaoud Z, Parham P. Dimorphism in the TCRgamma-chain repertoire defines 2 types of human immunity to Epstein-Barr virus. *Blood Adv*. (2020) 4:1198–205. doi: 10.1182/bloodadvances.2019001179

It should be:

95. Dimova T, Brouwer M, Gosselin F, Tassignon J, Leo O, Donner C, et al. Effector Vgamma9Vdelta2 T cells dominate the human fetal gammadelta T-cell repertoire. *Proc Natl Acad Sci U S A*. (2015) 112:E556-65.

102. Delfau MH, Hance AJ, Lecossier D, Vilmer E, Grandchamp B. Restricted diversity of V gamma 9-JP rearrangements in unstimulated human gamma/delta T lymphocytes. *Eur J Immunol*. (1992) 22:2437-43.

103. Arruda LCM, Gaballa A, Uhlin M. Graft gammadelta TCR Sequencing Identifies Public Clonotypes Associated with Hematopoietic Stem Cell Transplantation Efficacy in Acute Myeloid Leukemia Patients and Unravels Cytomegalovirus Impact on Repertoire Distribution. *J Immunol*. (2019) 202(6):1859-70

104. Djaoud Z, Parham P. Dimorphism in the TCRgamma-chain repertoire defines 2 types of human immunity to Epstein-Barr virus. *Blood Adv*. (2020) 4(7):1198-205.

The reference for “Designed specifically for promiscuous MHC class II binding, LLLLDRLNQLESKMS recognizes the important role CD4+ T cells play in immune responses to neoantigens” was erroneously written as:

104. Wells DK, van Buuren MM, Dang KK, Hubbard-Lucey VM, Sheehan KCF, Campbell KM, et al. Key Parameters of Tumor Epitope Immunogenicity Revealed Through a Consortium Approach Improve Neoantigen Prediction. *Cell*. (2020) 183:818–34 e13. doi: 10.1016/j.cell.2020.09.015

It should be:

105. Wells DK, van Buuren MM, Dang KK, Hubbard-Lucey VM, Sheehan KCF, Campbell KM, et al. Key Parameters of Tumor Epitope Immunogenicity Revealed Through a Consortium Approach Improve Neoantigen Prediction. *Cell*. (2020) 183(3):818–34 e13.

The reference for “These findings are highly consistent with studies by Greenshields-Watson et al, Gao et al, and Wang et al, who concordantly report a TCR-V segment bias in virus-responsive repertoires in infants, adults, and individuals with elite control of HIV, that is based on germline-encoded TCR-MHC contacts with complementary biochemical features of TCR and MHC molecules (62, 105, 106), which is indeed intriguing.” was erroneously written as:

62. Greenshields-Watson A, Attaf M, MacLachlan BJ, Whalley T, Rius C, Wall A, et al. CD4(+) T Cells Recognize Conserved Influenza A Epitopes through Shared Patterns of V-Gene Usage and Complementary Biochemical Features. *Cell Rep*. (2020) 32:107885. doi: 10.1016/j.celrep.2020.107885

105. Gao K, Chen L, Zhang Y, Zhao Y, Wan Z, Wu J, et al. Germline-Encoded TCR-MHC Contacts Promote TCR V Gene Bias in Umbilical Cord Blood T Cell Repertoire. *Front Immunol*. (2019) 10:2064. doi: 10.3389/fimmu.2019.02064

106. Wang Y, Tsitsiklis A, Devoe S, Gao W, Chu HH, Zhang Y, et al. Peptide Centric Vbeta Specific Germline Contacts Shape a Specialist T Cell Response. *Front Immunol*. (2022) 13:847092. doi: 10.3389/fimmu.2022.847092

It should be:

62. Greenshields-Watson A, Attaf M, MacLachlan BJ, Whalley T, Rius C, Wall A, et al. CD4(+) T Cells Recognize Conserved Influenza A Epitopes through Shared Patterns of V-Gene Usage and Complementary Biochemical Features. *Cell Rep*. (2020) 32:107885. doi: 10.1016/j.celrep.2020.107885

100. Wang Y, Tsitsiklis A, Devoe S, Gao W, Chu HH, Zhang Y, et al. Peptide Centric Vbeta Specific Germline Contacts Shape a Specialist T Cell Response. *Front Immunol*. (2022) 13:847092.

106. Gao K, Chen L, Zhang Y, Zhao Y, Wan Z, Wu J, et al. Germline-Encoded TCR-MHC Contacts Promote TCR V Gene Bias in Umbilical Cord Blood T Cell Repertoire. *Front Immunol*. (2019) 10:2064.

The reference for “Unconventional T cells, such as γδ T cells, recognize a broad spectrum of antigens, including non-peptide metabolites and lipids presented by non-classical MHC molecules. This enables them to rapidly respond in an innate-like manner, offering early defense that can contain infections before conventional T cells are fully activated” was erroneously written as:

126. Lv M, Zhang Z, Cui Y. Unconventional T cells in brain homeostasis, injury and neurodegeneration. *Front Immunol*. (2023) 14:1273459. doi: 10.3389/fimmu.2023.1273459

It should be:

129. Lv M, Zhang Z, Cui Y. Unconventional T cells in brain homeostasis, injury and neurodegeneration. *Front Immunol*. (2023) 14:1273459. doi: 10.3389/fimmu.2023.1273459

The reference for “(…) the present study: also CD1 or MHC class II presented ligands, promoting local immune responses, maintaining tissue homeostasis, and facilitating repair after injury (96,99, 127–129)” was erroneously written as:

96. Davey MS, Willcox CR, Joyce SP, Ladell K, Kasatskaya SA, McLaren JE, et al. Clonal selection in the human Vdelta1 T cell repertoire indicates gammadelta TCR-dependent adaptive immune surveillance. *Nat Commun*. (2017) 8:14760. doi: 10.1038/ncomms14760 doi: 10.1038/s41467-022-33488-2

99. Davey MS, Willcox CR, Hunter S, Kasatskaya SA, Remmerswaal EBM, Salim M, et al. The human Vdelta2(+) T-cell compartment comprises distinct innate-like Vgamma9(+) and adaptive Vgamma9(-) subsets. *Nat Commun*. (2018) 9:1760. doi: 10.1038/s41467-018-04076-0

127. Davey MS, Willcox CR, Baker AT, Hunter S, Willcox BE. Recasting Human Vdelta1 Lymphocytes in an Adaptive Role. *Trends Immunol*. (2018) 39:446–59. doi: 10.1016/j.it.2018.03.003

128. Mayassi T, Barreiro LB, Rossjohn J, Jabri B. A multilayered immune system through the lens of unconventional T cells. *Nature*. (2021) 595:501–10. doi: 10.1038/s41586-021-03578-0

129. Yang J, Yan H. Mucosal epithelial cells: the initial sentinels and responders controlling and regulating immune responses to viral infections. *Cell Mol Immunol*. (2021) 18:1628–30. doi: 10.1038/s41423-021-00650-7

It should be:

96. Davey MS, Willcox CR, Joyce SP, Ladell K, Kasatskaya SA, McLaren JE, et al. Clonal selection in the human Vdelta1 T cell repertoire indicates gammadelta TCR-dependent adaptive immune surveillance. *Nat Commun*. (2017) 8:14760. doi: 10.1038/ncomms14760 doi: 10.1038/s41467-022-33488-2

99. Davey MS, Willcox CR, Hunter S, Kasatskaya SA, Remmerswaal EBM, Salim M, et al. The human Vdelta2(+) T-cell compartment comprises distinct innate-like Vgamma9(+) and adaptive Vgamma9(-) subsets. *Nat Commun*. (2018) 9:1760. doi: 10.1038/s41467-018-04076-0

130. Davey MS, Willcox CR, Baker AT, Hunter S, Willcox BE. Recasting Human Vdelta1 Lymphocytes in an Adaptive Role. *Trends Immunol*. (2018) 9:446-59. doi: 10.1038/s41467-018-04076-0

131. Mayassi T, Barreiro LB, Rossjohn J, Jabri B. A multilayered immune system through the lens of unconventional T cells. *Nature*. (2021) 595:501-10. doi: 10.1038/s41586-021-03578-0

132. Yang J, Yan H. Mucosal epithelial cells: the initial sentinels and responders controlling and regulating immune responses to viral infections. *Cell Mol Immunol*. (2021) 18:1628–30. doi: 10.1038/s41423-021-00650-7

The reference for “Their capacity for rapid cytokine secretion and cytotoxicity complements physical epithelial defenses and innate immune sensing, enhancing early viral control” was erroneously written as:

130. Hackstein CP, Klenerman P. MAITs and their mates: “Innate-like” behaviors in conventional and unconventional T cells. *Clin Exp Immunol*. (2023) 213:1–9. doi: 10.1093/cei/uxad058

It should be:

133. Hackstein CP, Klenerman P. MAITs and their mates: “Innate-like” behaviors in conventional and unconventional T cells. *Clin Exp Immunol*. (2023) 213:1–9. doi: 10.1093/cei/uxad058

The reference for “Their relative resistance to exhaustion and pre-expanded tissue presence makes them attractive for therapies targeting infections, but also cancer, and inflammatory diseases” was erroneously written as:

131. Constantinides MG, Belkaid Y. Early-life imprinting of unconventional T cells and tissue homeostasis. *Science*. (2021) 374:eabf0095. doi: 10.1126/science.abf0095

It should be:

134. Constantinides MG, Belkaid Y. Early-life imprinting of unconventional T cells and tissue homeostasis. *Science*. (2021) 374:eabf0095. doi: 10.1126/science.abf0095

Also, reference “83. Benveniste PM, Roy S, Nakatsugawa M, Chen ELY, Nguyen L, Millar DG, et al. Generation and molecular recognition of melanoma-associated antigen-specific human gammadelta T cells. Sci Immunol. 2018;3(30)” was not cited in the article in all necessary instances. The citation has now been inserted in the section “γδ-T cells can recognize (peptide-loaded) MHC classes I and II” and should read: “In this context another report is significant (83) Vδ1+-γδ-T cells derived *in vitro* from human hematopoietic stem and progenitor cell (HSPC) can react with and expand in response to HLA-A2-presented melanoma antigen MART-1. The binding of the respective γδ-TCRs to MART-1-pMHC is less peptide-centric as compared to the interaction with a MART-1-specific αβ-TCR and it is speculated that MART-1 may act as specific stabilizer for the MHC for proper recognition by the respective γδ-TCRs. Intriguingly, the heteroclite peptide MART-1-(26-35) ELAGIGILTV is highly aliphatic: 8/10 amino acids are hydrophobic (83).”

There was a mistake in **Table 1** as published. The peptide sequence and length were incorrectly stated as the 16-mer LLLLDRLLNQLESKMS. This has been corrected to the accurate 15-mer vaccine peptide sequence, LLLLDRLNQLESKMS. The corrected **Table 1** appears below.

**Table 1:** Vaccine peptide LLLLDRLNQLESKMS.

Peptide	AA position in SARS-Cov-2 nuc	Peptide length
**L**LL**L**DR**L**NQLESKMS Pept.Pos:**1–4–7**(40)	221–235	15

G P A V I L M Aliphatic amino acids, increasing in hydrophobicity from left to right.

Amino acid positions that allow CD1 presentation of lipophilic peptides according to Castano etal. (40) are marked in bold and red.

There was a mistake in the caption of **Table 1** as published. The peptide sequence and length were incorrectly stated as the 16-mer LLLLDRLLNQLESKMS. This has been corrected to the accurate 15-mer vaccine peptide sequence, LLLLDRLNQLESKMS. The corrected caption of **Table 1** appears below.

“**Table 1** Vaccine peptide LLLLDRLNQLESKMS”.

There was a mistake in the caption of **Table 3** as published. The color of aliphatic (hydrophobic) amino acids was incorrectly labeled as red, the color of basic amino acids was incorrectly labeled as green. The corrected caption of **Table 3** appears below.

**Table 3:** Identical V segments usage in D1 and D2 by the most expanded TRAV and TRBV clonotypes (n≥1000).

Most highly expanded clonotypes carrying the most commonly used TRAV segments (left) and TRB segment (right)								
TRA				TRB				
Donor	CDR3	V	J	Donor	CDR3	V	D	J
D1	CATD**AR**T**G**RRALTF	TRAV17	TRAJ5	D1	CASS**LR**Q**GV**NTEAFF	TRBV18	TRBD1	TRBJ1-1
	CAA**V**N**AGG**TSYGKLTF	TRAV13-1	TRAJ52		CASS**P**S**PG**TDTQYF	TRBV18		TRBJ2-3
	CATD**RKG**SSASKIIF	TRAV17	TRAJ3		CASS**P**S**K**Q**GL**SPLHF	TRBV18	TRBD1	TRBJ1-6
	CAAS**PRIII**Q**GA**QKLVF	TRAV13-1	TRAJ54		CASS**P**T**GGAM**NEQFF	TRBV18	TRBD1	TRBJ2-1
	CAA**RGV**YNFNKFYF	TRAV13-1	TRAJ21		CASS**PKVG**T**KG**SNEQFF	TRBV18	TRBD2	TRBJ2-1
	CAA**RA**DKLIF	TRAV13-1	TRAJ34		CASS**PRL**T**G**S**G**NSPLHF	TRBV18	TRBD1	TRBJ1-6
	CAT**AT**NS**GG**YQKVTF	TRAV17	TRAJ13		CASS**PG**T**GA**YNEQFF	TRBV18	TRBD1	TRBJ2-1
	CAAS**IGI**YNFNKFYF	TRAV13-1	TRAJ21		CASS**PR**SS**GGA**NTGELFF	TRBV18	TRBD1	TRBJ2-2
	CAT**GLLI**Q**GA**QKLVF	TRAV17	TRAJ54		CASS**PRLAGGL**SSYNVQFF	TRBV18	TRBD2	TRBJ2-1
	CAS**RGA**SKIIF	TRAV13-1	TRAJ3		CASS**PRAG**T**G**SPGELFF	TRBV18	TRBD1	TRBJ2-2
	CAA**L**SYNQGGKLIF	TRAV13-1	TRAJ23		CASS**PG**STTGELFF	TRBV18		TRBJ2-2
	CATS**GG**NNRLAF	TRAV17	TRAJ7		CASS**PA**S**GRAGA**NVLTF	TRBV18	TRBD2	TRBJ2-6
	CAQSTYNQGGKLIF	TRAV13-1	TRAJ23		CASS**PAPG**QTNEQYF	TRBV18	TRBD1	TRBJ2-7
	CA**PR**N**G**S**GG**TSYGKLTF	TRAV13-1	TRAJ52		CASS**PGAR**NSPLHF	TRBV18	TRBD1	TRBJ1-6
	CA**F**T**G**NQFYF	TRAV17	TRAJ49		CASS**IK**T**G**NP**LV**NNEQFF	TRBV18	TRBD1	TRBJ2-1
D2	CAAS**ILGG**NQFYF	TRAV13-1	TRAJ49	D2	CASS**R**SP**G**Q**A**DTQYF	TRBV18		TRBJ2-3
	CIL**V**TS**G**TYKYIF	TRAV26-2	TRAJ40		CASS**PG**T**G**NTEAFF	TRBV18	TRBD1	TRBJ1-1
	CIL**RGG**NTGNQFYF	TRAV26-2	TRAJ49		CASS**I**SS**G**TSTDTQYF	TRBV18	TRBD2	TRBJ2-3
	CIL**RVWGF**GNEKLTF	TRAV26-2	TRAJ48		CASS**L**NY**VRG**QETQYF	TRBV18	TRBD1	TRBJ2-5
	CAAS**H**S**G**NTPLVF	TRAV13-1	TRAJ29		CASS**A**ST**GV**NEQFF	TRBV18	TRBD1	TRBJ2-1
	CILLS**G**NTPLVF	TRAV26-2	TRAJ29		CASS**PLG**TS**GR**NQETQYF	TRBV18	TRBD2	TRBJ2-5
	CAASTS**G**TYKYIF	TRAV13-1	TRAJ40		CASS**RG**T**GAL**NVLTF	TRBV18	TRBD1	TRBJ2-6
	CAASSN**G**GNQFYF	TRAV13-1	TRAJ49		CASS**PVKVAL**SGNTIYF	TRBV18		TRBJ1-3
	CIL**KGG**N**A**GNMLTF	TRAV26-2	TRAJ39		CASS**PV**P**IGVG**TDTQYF	TRBV18		TRBJ2-3
	CIL**R**D**WGG**EKLTF	TRAV26-2	TRAJ48		CASS**P**S**IV**S**GH**EQYF	TRBV18		TRBJ2-7
	CIL**R**D**VA**NFGNEKLTF	TRAV26-2	TRAJ48		CASS**PGLVG**SEQFF	TRBV18		TRBJ2-1
	CAASTS**G**TYKYIF	TRAV13-1	TRAJ40		CASS**K**S**PP**NTGELFF	TRBV18		TRBJ2-2
	CAAS**KPG**NNRKLI	TRAV13-1	TRAJ38		CASS**P**S**KIG**T**G**AYEQYF	TRBV18	TRBD1	TRBJ2-7
	CAA**RG**SDNRLAF	TRAV13-1	TRAJ7		CASS**RG**TNNSPLHF	TRBV18	TRBD2	TRBJ1-6
	CAAS**F**Y**GG**SQGNLIF	TRAV13-1	TRAJ42		CASS**RGR**QFEQYF	TRBV18		TRBJ2-7

TRAV segments (left) and TRB segment (right). Aliphatic (hydrophobic) amino acids given in green and bold, basic amino acids in red and bold. N nucleotide encoded aa are underlined.

“TRAV segments (left) and TRB segment (right). Aliphatic (hydrophobic) amino acids given in green and bold, basic amino acids in red and bold. N nucleotide encoded aa are underlined.”

There was a mistake in the caption of **Table 4** as published. The color of aliphatic (hydrophobic) amino acids was incorrectly labeled as red and bold, the color of basic amino acids was incorrectly labeled as green and bold. Additionally, the reference for the citation “iReceptor Scientific Gateway of the iReceptor platform” was erroneously written as:

**Table 4:** Overlap repertoire TRA, TRB, TRD overlap sequences lack or show only few N nucleotides.

Locus	Rearrange-ment	CDR3 V	…N…	J	V-segment	D segment	J segment	Np1_length	Np2_length	Donor	# of clones	CDR3 sequence	Public
TRA	VαCα	**CAG**	**RRR**	**QGAQKLVF**	TRAV35*01		TRAJ54*01	8		D1+D2	1 (D1)/2 (D2)	Ident.	+
	VαCα	**CAYRS**	**F**	**SGNTPLVF**	TRAV38-2/DV8*01		TRAJ29*01	5 (D1)/3 (D2		D1+D2	1 (D1)/1 (D2)	Con.	+
	VαCα	**CAYRS**		**YGGSQGNLIF**	TRAV38-2/DV8*01		TRAJ42*01	2 (D1)/0 (D2)		D1+D2	1 (D1)/1 (D2)	Con.	+
	VαCα	**CILR**	**V**	**NFGNEKLTF**	TRAV26-2*01		TRAJ48*01	2 (D1)/3 (D2)		D1+D2	1 (D1)/1 (D2)	Con.	+
	VαCα	**CILR**	**AP**	**FGNEKLTF**	TRAV26-2*01		TRAJ48*01	4 (D1+D2)		D1+D2	1 (D1)/1 (D2)	Ident.	+
	VαCα	**CA**	**V**	**TGNQFYF**	TRAV13-1*01		TRAJ49*01	5 (D1)		D1	1 (D1)	Con.	+
		**CAV**		**TGNQFYF**	TRAV21*01		TRAJ49*01	0 (D2)		D2	1 (D2)		+
	VαCα	**CAA**	**R**	**DTGRRALTF**	TRAV13-1*01		TRAJ5*01	3 (D1)		D1	1 (D1)	Con.	+
					TRAV13-1*02		TRAJ5*01	0 (D1+D2)		D1+D2	1 (D1)/1 (D2)	Con.	+
	VαCα	**CAAS**	**KA**	**GRRALTF**	TRAV13-1*01		TRAJ5*01	3 (D1)		D1	1 (D1)	Ident.	+
					TRAV13-1*02		TRAJ5*01	3 (D2)		D2	1 (D2)		+
	VαCα	**CAAS**		**TSGTYKYIF**	TRAV13-1*01		TRAJ40*01	0 (D1)		D1	1 (D1)	Con.	+
					TRAV13-1*02		TRAJ40*01	0/2 (D2)		D2	6 (D2)	Con.	+
	VαCα	**CAAS**		**NQGGKLIF**	TRAV13-1*01		TRAJ23*01	3 (D1)		D1	1 (D1)	Ident.	+
					TRAV13-1*02		TRAJ23*01	4 (D2)		D2	1 (D2)		+
	VαCα	**CA**	**L**	**RDDKIIF**	TRAV21*01		TRAJ30*01	0 (D1)		D1	1 (D1)	Con.	+
		**CAL**		**RDDKIIF**	TRAV19*01		TRAJ30*01	0 (D2)		D2	1 (D2)		+
	VαCα	**CAV**		**NSGGYQKVTF**	TRAV2*01		TRAJ13*02	1 (D1)		D1	2 (D1)	Con.	+
		**CAVN**		**SGGYQKVTF**	TRAV12-2*01		TRAJ13*02	0 (D2)		D2	1 (D2)	Con.	+
	VαCα	**CAA**	**P**	**NTGNQFYF**	TRAV29/DV5*04		TRAJ49*01	1 (D1)		D1	1 (D1)	Con.	+
					TRAV29/DV5*01		TRAJ49*01	3 (D2)		D2	1 (D2)		+
	VαCα	**C**	**D**	**NNNDMRF**	TRAV16*01		TRAJ43*01	0 (D1+D2)		D1+D2	1 (D1+D2)	Ident.	+
	VαCα	**CAA**	**L**	**DTGRRALTF**	TRAV13-1*01		TRAJ5*01	1 (D1+D2)		D1+D2	1 (D1+D2)	Con.	+
	VαJδCα	**VLPAL**	**L**	**SQTQLGSFPPLRP**	TRAV23/DV6*01	TRDD2*01	TRDJ4*01	**48** (D1+D2)	**8** (D1+D2)	D1+D2	1 (D1+D2)	Ident.	**no**
TRB	VβCβ	**CASSHGTSGR**	**L**	**GELFF**	TRBV3-1*01	TRBD2*02	TRBJ2-2*01	1	3 (D1)/2 (D2)	D1+D2	1 (D1)/1 (D2)	Con.	+
	VβCβ	**CASSP**	**GIS**	**SYEQYF**	TRBV18*01		TRBJ2-7*01	8 (D1)/9 (D2)		D1+D2	1 (D1)/1 (D2)	Con.	+
	VβCβ	**CASSL**	**SLS**	**TDTQYF**	TRBV11-2*03		TRBJ2-3*01	9 (D1)		D1	1 (D1)	Con.	+
					TRBV11-2*01		TRBJ2-3*01	7/6 (D2)		D2	2 (D2)	Con.	+
	VβCβ	**CASS**	**PSNG**	**AKNIQYF**	TRBV7-2*01		TRBJ2-4*01	11 (D1)/12(D2)		D1+D2	1 (D1)/1 (D2)	Con.	+
	VβCβ	**CASSPGTG**	**R**	**TGELFF**	TRBV18*01,	TRBD1*01	TRBJ2-2*01	1 (D1)/0/1 (D2)	3(D1)/2/3 (D2)	D1+D2	1 (D1)/2 (D2)	Con.	+
	VβCβ	**CASSP**	**SP**	**ANTGELFF**	TRBV18*01		TRBJ2-2*01	8 (D1+D2)		D1+D2	1 (D1)/1 (D2)	Con.	+
	VβCβ	**CASSLGG**		**GNQPQHF**	TRBV12-3*01	TRBD1*01	TRBJ1-5*01	0 (D1)/8 (D2)	1 (D1+D2)	D1+D2	1 (D1)/1 (D2)	Con.	+
	VβCβ	**CASRAG**	**A**	**NNEQFF**	TRBV7-9*01	TRBD2*01	TRBJ2-1*01	1 (D1)	3 (D2)	D1	1 (D1)	Con.	+
					TRBV7-9*03	TRBD2*01	TRBJ2-1*01	1 (D2)	5 (D2)	D2	6 (D2)	Con.	+
	VβCβ	**CASSL**	**S**	**ATNEKLF**	TRBV12-3*01		TRBJ1-4*01	5 (D1)/4 (D2)		D1+D2	1 (D1)/2 (D2)	Con.	+
TRD	VαCδ	**LPVSF**			TRAV12-1*01		TRAJ44*01	8 (D1+D2)		D1+D2	1 (D1+D2)	Ident.	+
							TRAJ20*01	8 (D1+D2)		D1+D2	1 (D1+D2)	Ident.	+
	VδCδ	**HCSHFLDPYSALCV**			TRAV36/DV7*01	TRDD2*01	TRDJ2*01	**58** (D1+D2)	**22** (D1+D2)	D1+D2	1 (D1	Ident.	**no**

CDR3 regions are displayed in bold. Aliphatic amino acids are given in green, basic amino acidsin red.Abbreviations in CDR3 sequence column: "ident." for identical sequences, "con." forconvergent sequences. Convergent sequences show the same nucleotide sequence but are derived fromrearrangments involving different VDJ segments. Screening for the public nature of a clonotype was performed using the iReceptor Scientific Gateway of the iReceptor platform (60).

**Table 5:** TRA and TRB top 10 clonotypes in D1 and D2.

Top 10 Clonotypes										
TRA					TRB					
Donor	CDR3	V	J	Public +/-	Donor	CDR3	V	D	J	Public +/-
D1	CAY**R**S**SRFG**NEKLTF	TRAV38-2/DV8*01	TRAJ48*01	+	D1	CATS**RVG**Q**GRLR**TGELFF	TRBV15*02	TRBD1*01	TRBJ2-2*01	-
	CATD**AR**T**GRR**ALTF	TRAV17*01	TRAJ5*01	+		CASS**RSSGIP**SYEQYF	TRBV5-1*01	TRBD2*02	TRBJ2-7*01	-
	CATD**RKG**SSASKIIF	TRAV17*01	TRAJ3*01	-		CASSYS**G**SNQPQHF	TRBV6-5*01	TRBD2*01	TRBJ1-5*01	+
	CAA**V**N**AGG**TSYGKLTF	TRAV13-1*02	TRAJ52*01	+		CASSE**SFM**AYEQYF	TRBV6-1*01		TRBJ2-7*01	-
	CAVS**V**T**GG**FKTIF	TRAV8-4*01	TRAJ9*01	+		CASSS**A**YEQYF	TRBV7-2*02		TRBJ2-7*01	+
	CAV**RLS**NTGNQFYF	TRAV41*01	TRAJ49*01	+		CS**GVGG**YEQYF	TRBV29-1*01	TRBD1*01	TRBJ2-7*01	+
	CAAS**MR**TSYGKLTF	TRAV23/DV6*01	TRAJ52*01	+		CASS**LR**Q**G**VNTEAFF	TRBV18*01	TRBD1*01	TRBJ1-1*01	+
	CAVQ**DTQG**TGNQFYF	TRAV20*02	TRAJ49*01	-		CASSD**SGGG**IQYF	TRBV6-1*01	TRBD2*01	TRBJ2-4*01	+
	CAAS**PRIII**QGAQKLVF	TRAV13-1*02	TRAJ54*01	-		CASS**LG**T**GR**TGELFF	TRBV5-5*01	TRBD1*01	TRBJ2-2*01	+
	CGGNNRLAF	TRAV12-2*01	TRAJ7*01	+		CAS**R**E**GA**TYEQYF	TRBV7-9*01	TRBD1*01	TRBJ2-7*01	+
D2	CATD**PS**AALIF	TRAV17*01	TRAJ15*01	-	D2	CASS**VRALA**GPDTQYF	TRBV9*01	TRBD2*02	TRBJ2-3*01	+
	CAAS**ILGG**NQFYF	TRAV13-1*02	TRAJ49*01	+		CAISE**GLAG**VYEQYF	TRBV10-3*02	TRBD2*02	TRBJ2-7*01	+
	CAY**R**S**Q**S**G**NTPLVF	TRAV38-2/DV8*01	TRAJ29*01	+		CASS**RSPGQ**ADTQYF	TRBV18*01		TRBJ2-3*01	+
	C**LGM**DT**GRR**ALTF	TRAV22*01	TRAJ5*01	+		CASS**PG**T**G**NTEAFF	TRBV18*01	TRBD1*01	TRBJ1-1*01	+
	CAY**RGRGGG**SNYKLTF	TRAV38-2/DV8*01	TRAJ53*01	+		CS**GWLAGSGG**ETQYF	TRBV29-1*01	TRBD2*02	TRBJ2-5*01	-
	CIL**V**TS**G**TYKYIF	TRAV26-2*01	TRAJ40*01	+		CAS**RAGA**NNEQFF	TRBV7-9*03	TRBD2*01	TRBJ2-1*01	+
	CA**PR**NDYKLSF	TRAV17*01	TRAJ20*01	+		CASS**I**SS**GT**STDTQYF	TRBV18*01	TRBD2*02	TRBJ2-3*01	+
	CATD**RGGGG**NKLTF	TRAV17*01	TRAJ10*01	+		CASS**LNYVRGQ**ETQYF	TRBV18*01	TRBD1*01	TRBJ2-5*01	-
	CAT**GH**T**GRR**ALTF	TRAV17*01	TRAJ5*01	+		CASS**AGLAG**EQYF	TRBV2*01	TRBD2*01	TRBJ2-7*01	+
	CIL**RGG**NT**G**NQFYF	TRAV26-2*01	TRAJ49*01	+		CASS**PLG**TS**GR**NQETQYF	TRBV18*01	TRBD2*02	TRBJ2-5*01	-

N-Nucleotide encoded amino acids are underlined and bold, alphatic amino acids are green andbold, basic amino acids are red and bold. Screening for the public nature of a clonotype wasperformed using the iReceptor Scientific Gateway of the iReceptor platform (60).

35. Kovjazin R, Volovitz I, Kundel Y, Rosenbaum E, Medalia G, Horn G, et al. ImMucin: a novel therapeutic vaccine with promiscuous MHC binding for the treatment of MUC1-expressing tumors. *Vaccine*. (2011) 29:4676–86. doi: 10.1016/j.vaccine.2011.04.103

It should be:

60. Corrie BD, Marthandan N, Zimonja B, Jaglale J, Zhou Y, Barr E, et al. iReceptor: A platform for querying and analyzing antibody/B-cell and T-cell receptor repertoire data across federated repositories. *Immunol Rev*. (2018) 284:24–41. doi: 10.1111/imr.2018.284.issue-1

The corrected caption of **Table 4** appears below.

“CDR3 regions are displayed in bold. Aliphatic amino acids are given in green, basic amino acids in red.”

Abbreviations in CDR3 sequence column: “ident.” for identical sequences, “con.” for convergent sequences. Convergent sequences show the same nucleotide sequence but are derived from rearrangements involving different VDJ segments. Screening for the public nature of a clonotype was performed using the iReceptor Scientific Gateway of the iReceptor platform (60).

There was a mistake in the caption of **Table 5** as published. The color of aliphatic (hydrophobic) amino acids was incorrectly labeled as red and bold, the color of basic amino acids was incorrectly labeled as green and bold. Additionally, the reference for the citation “iReceptor Scientific Gateway of the iReceptor platform” was erroneously written as:

35. Kovjazin R, Volovitz I, Kundel Y, Rosenbaum E, Medalia G, Horn G, et al. ImMucin: a novel therapeutic vaccine with promiscuous MHC binding for the treatment of MUC1-expressing tumors. *Vaccine*. (2011) 29:4676–86. doi: 10.1016/j.vaccine.2011.04.103

It should be:

60. Corrie BD, Marthandan N, Zimonja B, Jaglale J, Zhou Y, Barr E, et al. iReceptor: A platform for querying and analyzing antibody/B-cell and T-cell receptor repertoire data across federated repositories. *Immunol Rev*. (2018) 284:24–41. doi: 10.1111/imr.2018.284.issue-1

The corrected caption of **Table 5** appears below.

“N-Nucleotide encoded amino acids are underlined and bold, alphatic amino acids are green and bold, basic amino acids are red and bold. Screening for the public nature of a clonotype was performed using the iReceptor Scientific Gateway of the iReceptor platform (60).”

There was a mistake in the caption of **Table 8** as published. The color of aliphatic (hydrophobic) amino acids was incorrectly labeled as red, the color of basic amino acids was incorrectly labeled as green. The corrected caption of **Table 8** appears below.

**Table 8:** Exotic and hybrid γδTCRs including VδCα, VαJCδ, and atypical VδCδ clonotypes.

Exotic rearrangement	CDR3 sequence	Hydrophobic amino acids in CDR3	Public
Hybrid γδTCR (Vδ6/Cα)	**VLPALLSQTQLGSFPPLRP**	12/19	present in both donors, superpublic
Hybrid γδTCR (Vα12/J44/Cδ)	**LPVSF**	4/5	present in both donors, superpublic
Hybrid γδTCR (Vα12/J20/Cδ)	**LPVSF**	4/5	present in both donors, superpublic
γδTCR (Vδ7/Cδ)	**HCSHFLDPYSALC**	8/14	present in both donors, public

Aliphatic amino acids are marked in green, basic amino acids in red.CDR3 regions are given in bold.

“Aliphatic amino acids are marked in green, basic amino acids in red. CDR3 regions are given in bold.”

The original version of this article has been updated.

