# Supplementary material for: Promiscuous class II-binding SARS-CoV-2-nuc derived vaccine-peptide induced extensive conventional, innate and unconventional T cell responses
Source: Front Immunol. 2025 Nov 11;16:1676455. doi: 10.3389/fimmu.2025.1676455 (PMC12644032; doi:10.3389/fimmu.2025.1676455)
Supplement: Supplementary file 1 [file SupplementaryFile1.docx]

| Donor | # | CDR3 | V | J |
| --- | --- | --- | --- | --- |
| D1 | 1 | CAFGEPAHKAAGNKLTF | TRDV1*01 | TRAJ17*01 |
|  | 2 | CALGDRRDDKIIF | TRDV1*01 | TRAJ30*01 |
|  | 3 | CALGEAAAAGNKLTF | TRDV1*01 | TRAJ17*01 |
|  | 4 | CALGEGDRDDKIIF | TRDV1*01 | TRAJ30*01 |
|  | 5 | CALGEKDNNNDMRF | TRDV1*01 | TRAJ43*01 |
|  | 6 | CALGELGGDDKIIF | TRDV1*01 | TRAJ30*01 |
|  | 7 | CALGELGYSSASKIIF | TRDV1*01 | TRAJ3*01 |
|  | 8 | CALGELGYSSASKIIF | TRDV1*01 | TRAJ3*01 |
|  | 9 | CALGELRNTGFQKLVF | TRDV1*01 | TRAJ8*01 |
|  | 10 | CALGEPADKAAGNKLTF | TRDV1*01 | TRAJ17*01 |
|  | 11 | CALGEPAHKAAGNKLTF | TRDV1*01 | TRAJ17*01 |
|  | 12 | *convergent* | TRDV1*01 | TRAJ17*01 |
|  | 13 | *convergent* | TRDV1*01 | TRAJ17*01 |
|  | 14 | *convergent* | TRDV1*01 | TRAJ17*01 |
|  | 15 | CALGEPAHKAAVNKLTF | TRDV1*01 | TRAJ17*01 |
|  | 16 | CALGEPAHKATGNKLTF | TRDV1*01 | TRAJ17*01 |
|  | 17 | CALGEPPGKSTF | TRDV1*01 | TRAJ27*01 |
|  | 18 | CALGERAAGNKLTF | TRDV1*01 | TRAJ17*01 |
|  | 19 | CALGERAHDYKLSF | TRDV1*01 | TRAJ20*01 |
|  | 20 | CALGERTGRRALTF | TRDV1*01 | TRAJ5*01 |
|  | 21 | CALGERYGGSQGNLIF | TRDV1*01 | TRAJ42*01 |
|  | 22 | CALGESLYSSASKIIF | TRDV1*01 | TRAJ3*01 |
|  | 23 | CALGFTSGTYKYIF | TRDV1*01 | TRAJ40*01 |
|  | 24 | CALGGDFGNEKLTF | TRDV1*01 | TRAJ48*01 |
|  | 25 | CALGKPAHKAAGNKLTF | TRDV1*01 | TRAJ17*01 |
|  | 26 | CALGLRRALIF | TRDV1*01 | TRAJ15*01 |
|  | 27 | CALGLRSALIF | TRDV1*01 | TRAJ15*01 |
|  | 28 | CALGVLNYGGATNKL | TRDV1*01 | TRAJ32*02 |
|  | 29 | CALTLSGTYKYIF | TRDV1*01 | TRAJ40*01 |
|  | 30 | CALTYGGSQGNLIF | TRDV1*01 | TRAJ42*01 |
|  | 31 | CALWEPAHKAAGNKLTF | TRDV1*01 | TRAJ17*01 |
|  | 32 | CVLGEPAHKAAGNKLTF | TRDV1*01 | TRAJ17*01 |
| D2 | 1 | CALGESNGNQFYF | TRDV1*01 | TRAJ49*01 |
|  | 2 | CALGERAQGGKLIF | TRDV1*01 | TRAJ23*01 |
|  | 3 | CALGELAAGGFKTIF | TRDV1*01 | TRAJ9*01 |
|  | 4 | CALGELAAGGCKTIF | TRDV1*01 | TRAJ9*01 |
|  | 5 | CALGERYKAAGNKLTF | TRDV1*01 | TRAJ17*01 |
|  | 6 | CALGERYNAAGNKLTF | TRDV1*01 | TRAJ17*01 |

**Supplementary Table 1**

**Supplementary Table 1: Vδ1Cα Clonotypes.** N-Nucleotide encoded amino acids are underlined.

**Supplementary Table 2**

| Donor | # | CDR3 | V | D | J |
| --- | --- | --- | --- | --- | --- |
| D1 | 1 | CALGELPLRGKLIF | TRDV1*01 | TRDD2*01 | TRDJ1*01 |
|  | 2 | CALGELGGRITDKLIF | TRDV1*01 | TRDD3*01 | TRDJ1*01 |
|  | 3 | CALGERPSRRYYTDKLIF | TRDV1*01 |  | TRDJ1*01 |
|  | 4 | CALGETGRDWGYCDKLIF | TRDV1*01 | TRDD3*01 | TRDJ1*01 |
|  | 5 | CALGEQRSYWGTTDKLIF | TRDV1*01 | TRDD3*01 | TRDJ1*01 |
|  | 6 | CALDGRRRVPWRKRKLIF | TRDV1*01 |  | TRDJ1*01 |
|  | 7 | CALGVRSAGGFFTDKLIF | TRDV1*01 | TRDD3*01 | TRDJ1*01 |
|  | 8 | CALGYWAFPSTGGYKLIF | TRDV1*01 | TRDD3*01 | TRDJ1*01 |
|  | 9 | CALGELLGIRKEPELIF | TRDV1*01 | TRDD3*01 | TRDJ1*01 |
|  | 10 | CALGESWMRVRSDKLIF | TRDV1*01 |  | TRDJ1*01 |
|  | 11 | CALGDPRSTSYANWGPNDKLIF | TRDV1*01 | TRDD2*01 | TRDJ1*01 |
|  | 12 | CALGELDRLFPRWGIITDKLIF | TRDV1*01 | TRDD3*01 | TRDJ1*01 |
|  | 13 | CALGELYSSTGDNLGAKLIF | TRDV1*01 | TRDD3*01 | TRDJ1*01 |
|  | 14 | CALGTIPLGGGFRYTDKLIF | TRDV1*01 | TRDD3*01 | TRDJ1*01 |
|  | 15 | CALGEHIFLPLGDYTDKLIF | TRDV1*01 | TRDD3*01 | TRDJ1*01 |
|  | 16 | CALGELFLIWGIAYTDKLIF | TRDV1*01 | TRDD3*01 | TRDJ1*01 |
|  | 17 | CALGELGDYLLGLIF | TRDV1*01 | TRDD3*01 | TRDJ1*01 |
|  | 18 | CALGESSSYPYWGTPPLYTDKLIF | TRDV1*01 | TRDD3*01 | TRDJ1*01 |
|  | 19 | CALGELRDWASPTNWGYGAPKLIF | TRDV1*01 | TRDD3*01 | TRDJ1*01 |
|  | 20 | CALGENFGGLPTVLGDTNTDKLIF | TRDV1*01 | TRDD3*01 | TRDJ1*01 |
|  | 21 | CALGELYVYQTTLRLGDTPDKLIF | TRDV1*01 | TRDD3*01 | TRDJ1*01 |
|  | 22 | CALGEVFPPVGEYTDKLIF | TRDV1*01 | TRDD3*01 | TRDJ1*01 |
|  | 23 | CALGESLLGDNRKTDKLIF | TRDV1*01 | TRDD3*01 | TRDJ1*01 |
|  | 24 | CALGAPFLLELRTWDKLIF | TRDV1*01 | TRDD2*01 | TRDJ1*01 |
|  | 25 | GPKDADKLIF | TRDV1*01 | TRDD3*01 | TRDJ1*01 |
|  | 26 | CALGELGTWEVGDSSTDKLIF | TRDV1*01 | TRDD3*01 | TRDJ1*01 |
|  | 27 | CALGVVKPSYFTWRGPRKLIF | TRDV1*01 | TRDD2*01 | TRDJ1*01 |
|  | 28 | CALGETWGWGIRPPYTDKLIF | TRDV1*01 | TRDD3*01 | TRDJ1*01 |
|  | 29 | CALGEGPTGGYRTGSTDKLIF | TRDV1*01 | TRDD3*01 | TRDJ1*01 |
|  | 30 | CALGDRNFLPSYWGITYTDKLIF | TRDV1*01 | TRDD3*01 | TRDJ1*01 |
|  | 31 | CALEKLQTVRPTVLGDVPDKLIF | TRDV1*01 | TRDD3*01 | TRDJ1*01 |
|  | 32 | CALGERKWALGDTVTPYTDKLIF | TRDV1*01 | TRDD3*01 | TRDJ1*01 |
|  | 33 | CALGEPQEPFLFGLLGDTPTDKLIF | TRDV1*01 | TRDD3*01 | TRDJ1*01 |
|  | 34 | CALGEQLTFLVYWGMGTPYTDKLIF | TRDV1*01 | TRDD3*01 | TRDJ1*01 |
|  | 35 | CALGELGILPGPLGDTRISPAIKLIF | TRDV1*01 | TRDD3*01 | TRDJ1*01 |
|  | 36 | CALGERALLMTRYDLLGDTLRFAGTTDKLIF | TRDV1*01 | TRDD3*01 | TRDJ1*01 |
|  | 37 | CALLFLPTRSVTAQLFF | TRDV1*01 | TRDD2*01 | TRDJ2*01 |
|  | 38 | CALGEIFLRGIRKGALTAQLFF | TRDV1*01 | TRDD3*01 | TRDJ2*01 |
|  | 39 | CALGEIPTLGGWGKTAQLFF | TRDV1*01 | TRDD3*01 | TRDJ2*01 |
|  | 40 | CALGELSPHWGILVTAQLFF | TRDV1*01 | TRDD3*01 | TRDJ2*01 |
|  | 41 | TGSKVFRNDACGSALTAQLFF | TRDV1*01 | TRDD2*01 | TRDJ2*01 |
|  | 42 | CALGEISGTSSWDTRQMF | TRDV1*01 |  | TRDJ3*01 |
|  | 43 | CALGEGVRMEYWGIRSSWDTRQMF | TRDV1*01 | TRDD3*01 | TRDJ3*01 |
|  | 44 | CALGPPHPTTGGFSWDTRQMF | TRDV1*01 | TRDD3*01 | TRDJ3*01 |
| D2 | 1 | CALGEKGGGPSGTDKLIF | TRDV1*01 | TRDD3*01 | TRDJ1*01 |
|  | 2 | CALGETSYPRSYWGLYTDKLIF | TRDV1*01 | TRDD3*01 | TRDJ1*01 |
|  | 3 | *convergent* | TRDV1*01 | TRDD3*01 | TRDJ1*01 |
|  | 4 | CALGELVSRRRWGIRGGELIF | TRDV1*01 | TRDD3*01 | TRDJ1*01 |
|  | 5 | CALGIPKALHPVWGIRRTDKLIF | TRDV1*01 | TRDD3*01 | TRDJ1*01 |
|  | 6 | CALGIPKALHPVWVIRRTDKLIF | TRDV1*01 | TRDD3*01 | TRDJ1*01 |
|  | 7 | CALGELAFLYVYWGIRPYTDKLIF | TRDV1*01 | TRDD3*01 | TRDJ1*01 |
|  | 8 | CALGKGGRIGDKLIF | TRDV1*01 | TRDD1*01 | TRDJ1*01 |
|  | 9 | CALGTLWGIRFGYRGQLFF | TRDV1*01 | TRDD3*01 | TRDJ2*01 |

**Supplementary Table 2: Vδ1Cδ Clonotypes.** N-Nucleotide encoded amino acids are underlined.

**Supplementary Table 3**

| **Top 10 Clonotypes** | | | | |
| --- | --- | --- | --- | --- |
| **Vδ2** | | | | |
|  | **CDR3** | **D** | **J** | **Public**  **+/-** |
| **D1** | CACDTLLGDTYTDKLIF | TRDD3*01 | TRDJ1*01 | + |
|  | CACDRVPLGDREPDKLIF | TRDD3*01 | TRDJ1*01 | - |
|  | CACDILGDTVDSSWDTRQMF | TRDD3*01 | TRDJ3*01 | - |
|  | CACDTLGEGRIDKLIF | TRDD3*01 | TRDJ1*01 | - |
|  | CACDSTGGSWDTRQMF | TRDD3*01 | TRDJ3*01 | + |
|  | CACVPLPTTGGYAESAQLFF | TRDD3*01 | TRDJ2*01 | - |
|  | CACESLLRGLGDTDKLIF | TRDD3*01 | TRDJ1*01 | - |
|  | CACDTLTPPGRETDKLIF |  | TRDJ1*01 | - |
|  | CACDTLLGDTGSSDKLIF | TRDD3*01 | TRDJ1*01 | - |
|  | CACDSVLGDYFTAQLFF | TRDD3*01 | TRDJ2*01 | - |
| **D2** | CACDALRDTDKLIF |  | TRDJ1*01 | - |
|  | CACDTAAGGHRSSWDTRQMF | TRDD3*01 | TRDJ3*01 | - |
|  | CACDQLETPTDKLIF |  | TRDJ1*01 | - |
|  | CACDTVLGDTRRGHTDKLIF | TRDD3*01 | TRDJ1*01 | - |
|  | CACDPVLGDTTYTDKLIF | TRDD3*01 | TRDJ1*01 | + |
|  | CACDTVLGIRYTDKLIF | TRDD3*01 | TRDJ1*01 | - |
|  | CACDTVTGGGSAWDTRQMF | TRDD3*01 | TRDJ3*01 | - |
|  | CACDTMGYTDKLIF | TRDD3*01 | TRDJ1*01 | + |
|  | CACDTVGLGDKLIF |  | TRDJ1*01 | - |
|  | CACDTVMGTGGSGTDKLIF | TRDD3*01 | TRDJ1*01 | - |

**Supplementary Table 3: Top 10 Vδ2 Clonotypes.** Top 10 Vδ2 clonotypes and rearrangement of both donors. Screening for the public nature of a clonotype was performed using the iReceptor Scientific Gateway of the iReceptor platform^39^.
